# Hepatic miR-378 modulates serum cholesterol levels by regulating hepatic bile acid synthesis

**DOI:** 10.7150/thno.53624

**Published:** 2021-02-25

**Authors:** Chao Sun, Wei Liu, Zhiqiang Lu, Yan Li, Shengnan Liu, Zhili Tang, Ying Yan, Zhiyang Li, Hua Feng, Duo Zhang, Yun Liu, Zhong-Ze Fang, Changtao Jiang, Qiurong Ding, Jingjing Jiang, Hao Ying

**Affiliations:** 1 CAS Key Laboratory of Nutrition, Metabolism and Food Safety, Shanghai Institute of Nutrition and Health, University of Chinese Academy of Sciences, Chinese Academy of Sciences, Shanghai 200031, China.; 2 Department of Endocrinology and Metabolism, Zhongshan Hospital, Fudan University, Shanghai 200032, China.; 3 State Key Laboratory of Food Science and Technology, School of Food Science and Technology, Jiangnan University, Wuxi 214122, China.; 4 Omics Core, Bio-Med Big Data Center, CAS-MPG Partner Institute for Computational Biology, Shanghai Institutes for Biological Sciences, Chinese Academy of Sciences, Shanghai 200031, China.; 5 Division of Pulmonary and Critical Care Medicine, Department of Medicine, Boston University Medical Campus, Boston, MA 02118, USA.; 6 Shanghai Xuhui Central Hospital, Shanghai Clinical Center, Chinese Academy of Sciences, Shanghai 200031, China.; 7 Department of Toxicology and Sanitary Chemistry, School of Public Health, Tianjin Medical University, Tianjin 300070, China.; 8 Department of Physiology and Pathophysiology, School of Basic Medical Sciences, Peking University, and the Key Laboratory of Molecular Cardiovascular Science, Ministry of Education, Beijing 100191, China.; 9 Key Laboratory of Food Safety Risk Assessment, Ministry of Health, Beijing 100021, China.

**Keywords:** mmu-miR-378-3p, cholesterol, bile acid, MAFG, thyroid hormone.

## Abstract

**Rationale:** An improved understanding of thyroid hormone (TH) action on cholesterol metabolism will facilitate the identification of novel therapeutic targets for hypercholesterolemia. TH-regulated microRNAs (miRNAs) have been implicated in TH-controlled biological processes; however, whether and how TH-regulated miRNAs mediate the cholesterol-lowering effect of TH remains unclear. Our aim was to identify TH-regulated microRNAs that have cholesterol-lowering effects and explore the underlying mechanism.

**Method:** Microarray and RNA-seq were performed to identify TH-regulated microRNAs and the genes regulated by mmu-miR-378-3p (miR-378) in the liver of mice, respectively. Recombinant adenoviruses encoding miR-378, *Mafg*, and shRNA for* Mafg*, antagomiR-378, liver-specific miR-378 transgenic mice, and miR-378 knockout mice were employed to investigate the roles of hepatic miR-378 and MAFG in cholesterol and bile acid homeostasis. The levels of bile salt species were determined by using UFLC-Triple-time of flight/MS.

**Results:** Here, we show that hepatic miR-378 is positively regulated by TH. Transient overexpression of miR-378 in the liver of mice reduces serum cholesterol levels, accompanied with an increase in the expression of key enzymes in primary bile acid synthetic pathways and corresponding increases in biliary and fecal bile acid levels. Consistently, liver-specific miR-378 transgenic mice with moderate overexpression of hepatic miR-378 display decreased serum cholesterol levels and resistance to diet-induced hypercholesterolemia, while mice lacking miR-378 exhibit defects in bile acid and cholesterol homeostasis. Mechanistically, hepatic miR-378 regulates the expression of key enzymes in both classic and alternative bile acid synthetic pathways through MAFG, a transcriptional repressor, thereby modulating bile acid and cholesterol metabolism.

**Conclusions:** TH-responsive hepatic miR-378 is capable of modulating serum cholesterol levels by regulating both the classic and alternative BA synthetic pathways. Our study not only identifies a previously undescribed role of hepatic miR-378 but also provides new cholesterol-lowering approaches.

## Introduction

Cholesterol is an essential structural component of cell membranes and a precursor for steroid hormone and bile acid (BA) synthesis. Maintaining whole-body cholesterol homeostasis is crucial because high plasma cholesterol levels increase the risk of atherosclerotic cardiovascular disease 1, 2. Cholesterol homeostasis is maintained by the cooperative regulation of cholesterol uptake and *de novo* synthesis together with cholesterol catabolism to BAs 3, 4. Hepatic BA synthesis is the primary metabolic pathway for cholesterol catabolism in humans. Approximately 500 mg of cholesterol is converted into BAs every day in the adult human liver cells, which will be ultimately excreted from the body 5-7. Thus, strategies that promote the conversion of cholesterol to BAs should be valuable to lower plasma cholesterol levels, thereby preventing cardiovascular disease.

Thyroid hormone (TH), which has profound effects on cholesterol homeostasis, is well-known for its cholesterol-lowering property 8, 9. Hyperthyroidism is associated with increased hepatic cholesterol catabolism and reduced serum cholesterol levels, while hypothyroidism is associated with reduced cholesterol clearance and elevated serum cholesterol levels 10, 11. Based on improved mechanistic understanding of TH actions, great progress has been made in developing TH analogues to avoid undesirable side effects for the treatment of hypercholesterolemia and the prevention of atherosclerotic cardiovascular disease 12-15. One of the proposed mechanisms accounting for the cholesterol-lowering effect of TH is that TH increases the conversion of cholesterol into BAs 12, 13. Thus, we hypothesize that a better understanding of the TH action on hepatic BA synthesis will improve our likelihood of identifying novel therapeutic targets for the treatment of hypercholesterolemia.

There are two major BA synthetic pathways, classic and alternative BA synthetic pathways, which involve more than 17 enzymes 5-7. The classical pathway is initiated by CYP7A1, while the alternative pathway is initiated by CYP27A1. Primary BAs produced in humans are cholic acid (CA) and chenodeoxycholic acid (CDCA), while rodents produce CA and muricholic acids (MCAs). Of note, the classical pathway generates both CA and CDCA/MCAs, while the alternative pathway predominantly generates CDCA/MCAs. The ratio between CA and CDCA/MCAs is modulated by CYP8B1, which participates in CA synthesis. The expression of a set of key enzymes in BA synthesis is finely modulated by TH, emphasizing the importance of TH in maintaining normal BA homeostasis 16-20. Recently, TH-regulated microRNAs (miRNAs) have been implicated in TH-controlled physiological and pathological processes 21-23. These recent findings together with the fact that TH has the cholesterol-lowering effect prompted us to examine the role of TH-regulated miRNAs in hepatic BA synthesis and cholesterol homeostasis.

Here, we demonstrate that hepatic miR-378 is positively regulated by TH. Mice with hepatic mmu-miR-378-3p (miR-378) overexpression not only have reduced serum cholesterol levels but also have a BA phenotype that includes increased expression of CYP7B1, CYP8B1 and CYP27A1 in primary BA biosynthetic pathway and corresponding increases in BA levels in the bile and feces. Mechanistically, hepatic miR-378 regulates the expression of these key enzymes through its target gene, V-Maf Avian Musculoaponeurotic Fibrosarcoma Oncogene Homolog G (MAFG), which is a transcriptional repressor of BA synthetic genes 24. Notably, moderate overexpression of hepatic miR-378 is sufficient to lower serum cholesterol levels and prevent from diet-induced hypercholesterolemia, while mice lacking miR-378 display defects in BA and cholesterol homeostasis. Thus, our study not only defines hepatic miR-378 as an important regulator in BA and cholesterol metabolism but also provides new cholesterol-lowering approaches.

## Results

### TH-responsive hepatic miR-378 controls serum cholesterol levels

**Figure 1:**
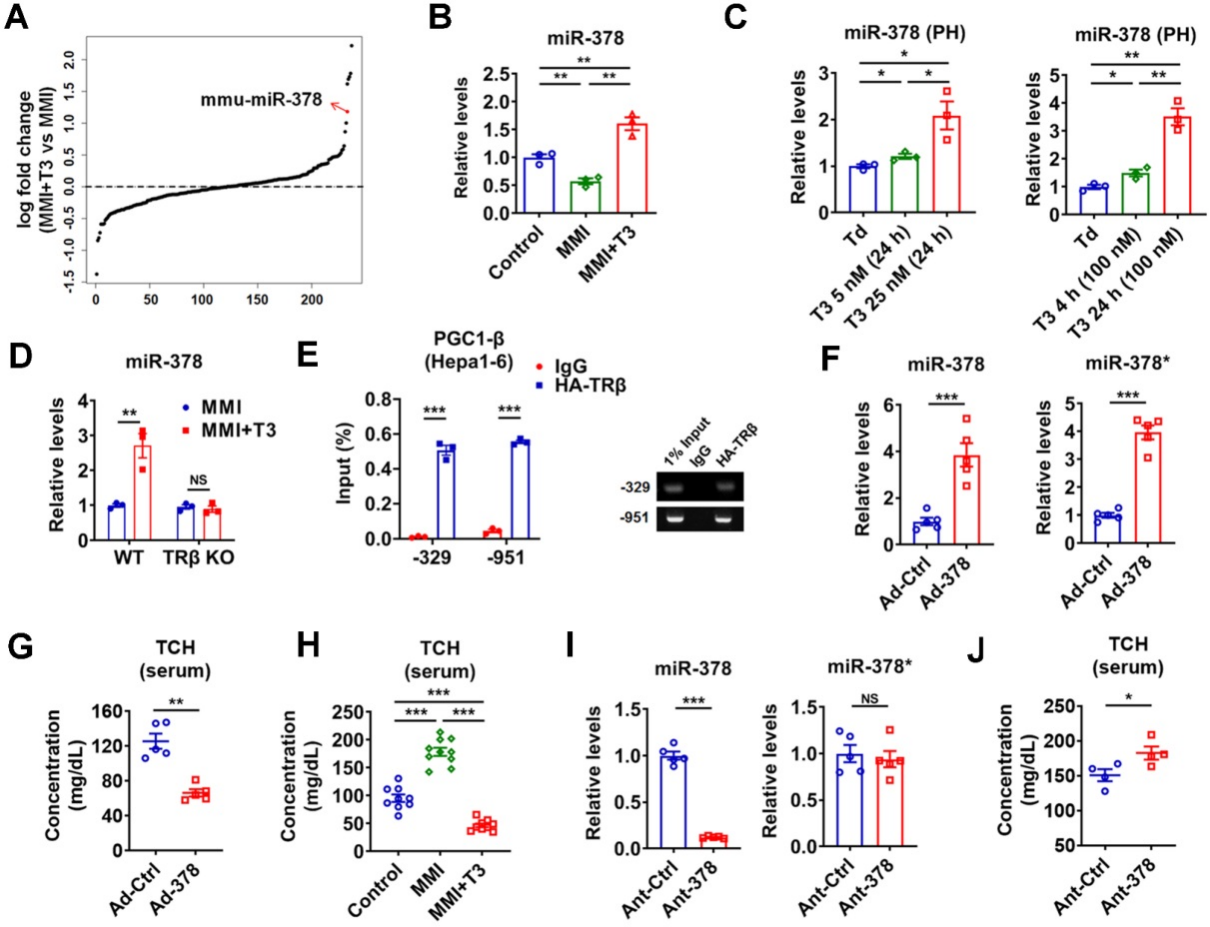
** TH-responsive hepatic miR-378 modulates serum cholesterol levels.** (A) Fold enrichment analysis of microarray data in the liver of hypothyroid mice before (MMI) and after administration of a single dose of T3 (MMI+T3). The fold enrichment of miR-378 reached 2.27 X, ranking 5^th^ among all the differentially expressed miRNAs. (B) Relative levels of miR-378 in the liver of euthyroid (control), hypothyroid (MMI) and hyperthyroid (MMI+T3) mice (n = 3). (C) Relative levels of miR-378 in the primary murine hepatocytes (PHs) cultured in the absence of T3 (Td, T3 deficient) or in the presence T3 (n = 3). (D) Relative levels of hepatic miR-378 in hypothyroid and hyperthyroid wild-type (WT) or TRβ knockout (TRβ KO) mice (n = 3). (E) ChIP analysis of the recruitment of TRβ to the proximal promoter regions of PGC1-β as indicated in Hepa1-6 cells transfected with HA-TRβ. qPCR products were analyzed by agarose gel electrophoresis as indicated. (F and G) Relative levels of hepatic miR-378 and miR-378* (F, n = 5) and serum total cholesterol levels (TCH) (G, n = 5) in mice infected with Ad-Ctrl or Ad-378. (H) Serum total cholesterol (TCH) levels of euthyroid (control), hypothyroid (MMI) and hyperthyroid (MMI+T3) mice (n = 9-10). (I and J) Relative levels of hepatic miR-378 and miR-378* (I, n = 5) and serum total cholesterol (TCH) levels (J, n = 4) in mice treated with Ant-Ctrl or Ant-378. Means ± SEM are shown. *p < 0.05; **p < 0.01; ***p < 0.001.

Given that liver is the major target that mediates the cholesterol-lowering effect of TH 13, we focused on hepatic TH-responsive miRNAs that may be involved. To identify hepatic miRNAs that have maximal T3 responsiveness, microarray analysis was carried out to compare the miRNA expression profiles in hypothyroid mice before and after injection of a single dose of T3 (GSE139961). We found that none of the 16 top-ranked differentially expressed (> 1.5-fold) miRNAs had been previously reported to have cholesterol-lowering effects (Table S1 and Figure S1A). Notably, excluding the let-7 family members, miR-378 ranked the 1^st^ among the rest of differentially expressed miRNAs in the liver of hypothyroid mice after T3 injection, indicating that miR-378 might be crucial for the regulatory role of TH (Figure 1A and Figure S1A). As miR-378 has been implicated in multiple metabolic processes 25-29, we then chose miR-378 for further investigation. First of all, the expression of hepatic miR-378 was examined in the liver of mice under different thyroid status. In line with the microarray data, hepatic miR-378 levels were significantly decreased in hypothyroid mice as compared to euthyroid mice, while hepatic miR-378 levels were significantly increased in hyperthyroid mice as compared to either euthyroid or hypothyroid mice (Figure 1B and Figure S1B). Consistently, T3 treatment increased the levels of miR-378 in primary murine hepatocytes in a dose- and time-dependent manner, respectively (Figure 1C), indicating that T3 regulates hepatic miR-378 expression in a cell-autonomous manner. Notably, similar expression pattern was also observed for PGC-1β, the miR-378 host gene, and mmu-miR-378-5p (miR-378*) both *in vivo* and *in vitro* (Figure S1C-D). To further explore the role of TH in regulating hepatic miR-378, we employed mice lacking thyroid hormone receptor β (TRβ) 30, the major TR isoform in liver. Interestingly, we found that the regulation of hepatic miR-378 by T3 was abolished in hypothyroid mice lacking TRβ (Figure 1D), suggesting that T3 might regulate the expression of hepatic miR-378 through TRβ-mediated transcriptional regulation. Accordingly, the results of chromatin immunoprecipitation (ChIP) assay showed that TRβ could be recruited to the promoter regions of *Pgc-1β* (Figure 1E). Collectively, these results indicate that TH might regulate the expression of hepatic PGC-1β and its intronic miR-378 via its nuclear receptor at transcriptional level.

To test whether hepatic miR-378 is capable of regulating cholesterol metabolism, adenoviral miR-378/378* (Ad-378) was used to achieve a 4-fold specific overexpression of miR-378 in the liver of wild-type mice without any treatment (Figure 1F and Figure S1E). Interestingly, similar to what we observed for T3 treatment, Ad-378 infection could significantly decrease the total serum cholesterol levels but not the hepatic cholesterol levels in mice (Figure 1G-H and Figure S1F). Furthermore, when low dosage of synthetic antagomir specific for miR-378 (Ant-378) was used to reduce the miR-378 but not miR-378* levels in the liver but not in the fat or muscle of mice, the serum cholesterol levels were significantly increased (Figure 1I-J and Figure S1G). To be noted, infection with either Ad-Ctrl or Ant-Ctrl did not affect the hepatic expression of miR-378 and miR-378* and the serum cholesterol levels (Figure S1H). Collectively, these data suggest that hepatic miR-378 is able to regulate serum cholesterol levels.

### Hepatic miR-378 regulates BA biosynthetic pathway in the liver

**Figure 2:**
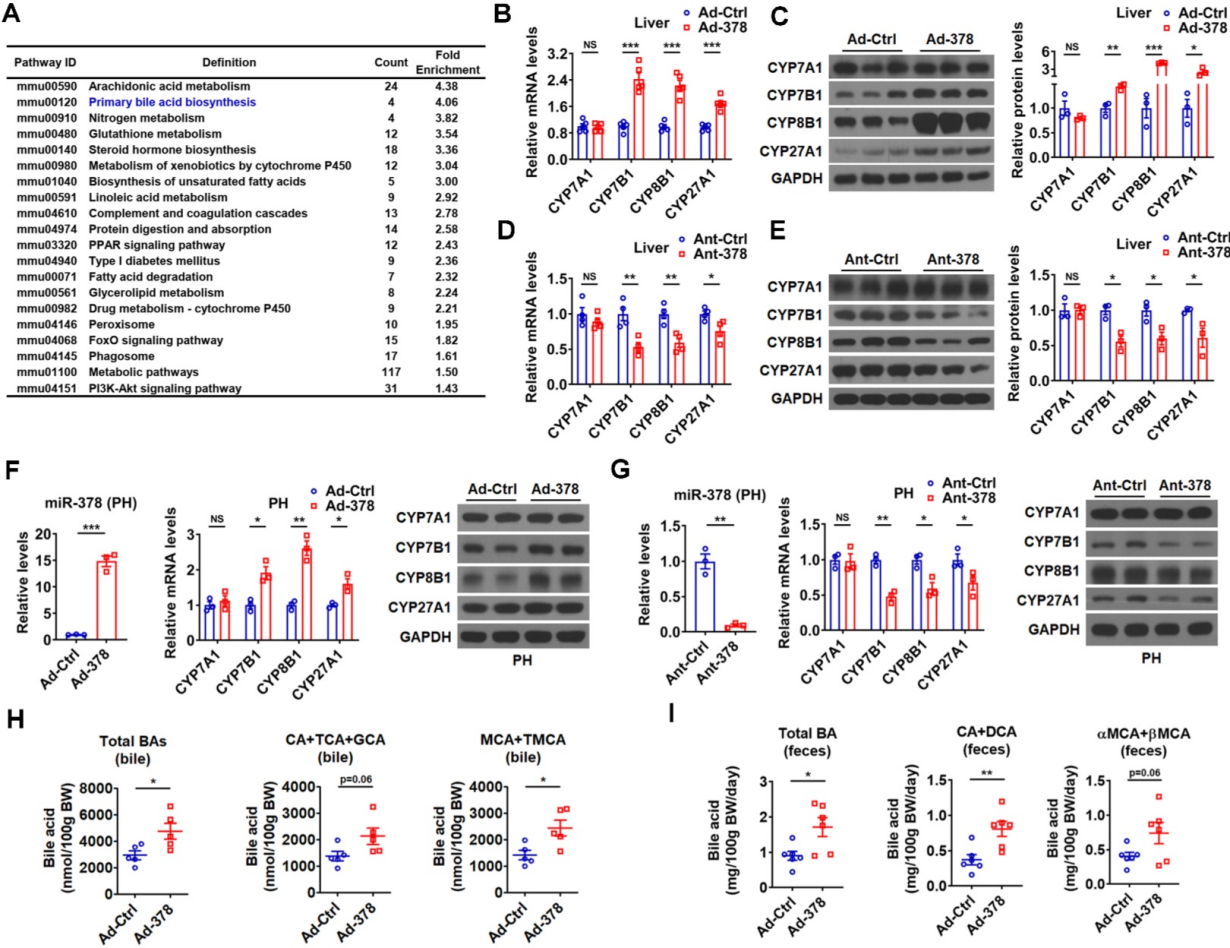
** Hepatic miR-378 regulates the synthesis and excretion of BAs.** (A) KEGG pathway analysis of differentially expressed genes identified by RNA-seq in the liver of mice infected with Ad-378 (n = 3). (B and C) Relative mRNA levels (B, n = 5), western blot and densitometry analysis (C, n = 3) of key enzymes of BA synthetic pathway in the liver of mice infected with Ad-Ctrl or Ad-378. (D and E) Relative mRNA levels (D, n = 4), western blot and densitometry analysis (E, n = 3) of key enzymes of BA synthetic pathway in the liver of mice treated with Ant-Ctrl or Ant-378. (F and G) Relative levels of miR-378 and mRNA levels of key enzymes of BA synthetic pathway (n = 3), and western blot analysis of key enzymes of BA synthetic pathway in the primary murine hepatocytes (PHs) infected with Ad-378 (F), or treated with Ant-378 (G). (H and I) The levels of total and major BAs in the bile (H, n = 5) and feces (I, n = 6) of mice infected with Ad-Ctrl or Ad-378 as indicated. Means ± SEM are shown. *p < 0.05; **p < 0.01; ***p < 0.001.

To understand the molecular mechanism underlying the effects of hepatic miR-378 on serum cholesterol levels, we performed RNA-seq analysis to identify genes regulated by miR-378 (Figure S2A and GSE139830). KEGG pathway analysis of differentially expressed genes in the liver of mice infected with Ad-378 revealed that miR-378 might play a regulatory role in primary BA biosynthesis (Figure 2A). We then determined the expression of key enzymes in BA synthetic pathway after hepatic miR-378 overexpression or inhibition. Interestingly, the mRNA and protein levels of CYP7A1, were not altered in the liver of mice after either Ad-378 infection or Ant-378 treatment (Figure 2B-E). In contrast, the mRNA and protein levels of hepatic CYP7B1, CYP8B1, and CYP27A1 were all increased by Ad-378 infection and decreased by Ant-378 administration, respectively (Figure 2B-E). The effects of Ad-378 infection on the protein levels of CYP7A1, CYP7B1, and CYP8B1 were further verified by ELISA (Figure S2B). Similar results were obtained in primary murine hepatocytes after Ad-378 infection or Ant-378 transfection (Figure 2F-G), suggesting that miR-378 regulates hepatic CYP7B1, CYP8B1, and CYP27A1 expression in a cell-autonomous manner. Since synthetic agomir for miR-378* (AgomiR-378*) transfection could not affect the mRNA expression of CYP7B1, CYP8B1, and CYP27A1, we speculated that miR-378 but not miR-378* contributes to the effect of Ad-378 infection on the expression of these enzymes (Figure S2C). In agreement with the finding that overexpression of hepatic miR-378 had no effect on CYP7A1 expression levels, the serum 7α-hydroxy-4-cholesten-3-one (C4) levels, which normally reflect the activity of CYP7A1 31, were not altered after Ad-378 infection (Figure S2D). Notably, Ad-378 infection increased the total amount of BAs in the bile of gallbladders but not in the liver and serum (Figure 2H and Figure S2E). Given that the hepatic expression of CYP7B1, CYP8B1, and CYP27A1 but not CYP7A1 was increased by Ad-378 infection, the increased BA levels in the bile of gallbladders implies that miR-378 overexpression might enhance the conversion of cholesterol to BAs through CYP7B1, CYP8B1, and CYP27A1, but not CYP7A1.

Since the rodents produce CA and MCAs, predominantly βMCA as primary BAs 32, we then analyzed both the amount and percentage of these primary BAs and their major metabolites. The levels of CA, taurine-conjugated CA (TCA), and glycine-conjugated CA (GCA) (CAs: CA+TCA+GCA) were tended to be increased in the bile of mice infected with Ad-378, while the levels of MCA (αMCA and βMCA) and taurine-conjugated MCA (TMCA: TαMCA and TβMCA) (MCAs: MCA+TMCA) were significantly increased in the bile of mice infected with Ad-378 (Figure 2H). Notably, the percentage of major biliary BAs (CAs and MCAs) was not significantly altered in the bile after Ad-378 administration (Figure S2F). As expected, the amount of total and major fecal BAs, including CA and its metabolite deoxycholic acid (DCA) (CA+DCA), as well as αMCA and βMCA (αMCA+βMCA) in the feces of Ad-378 infected mice were significantly or tended to be increased (Figure 2I), indicating that the fecal BA excretion was increased after Ad-378 infection. Notably, the food intake and feces output were not altered by Ad-378 infection (Figure S2G). Collectively, our data indicate that the increased hepatic BA synthesis and BA excretion might contribute to the decreased serum cholesterol levels after hepatic miR-378 overexpression.

### MAFG is a direct target gene of miR-378 in liver

**Figure 3:**
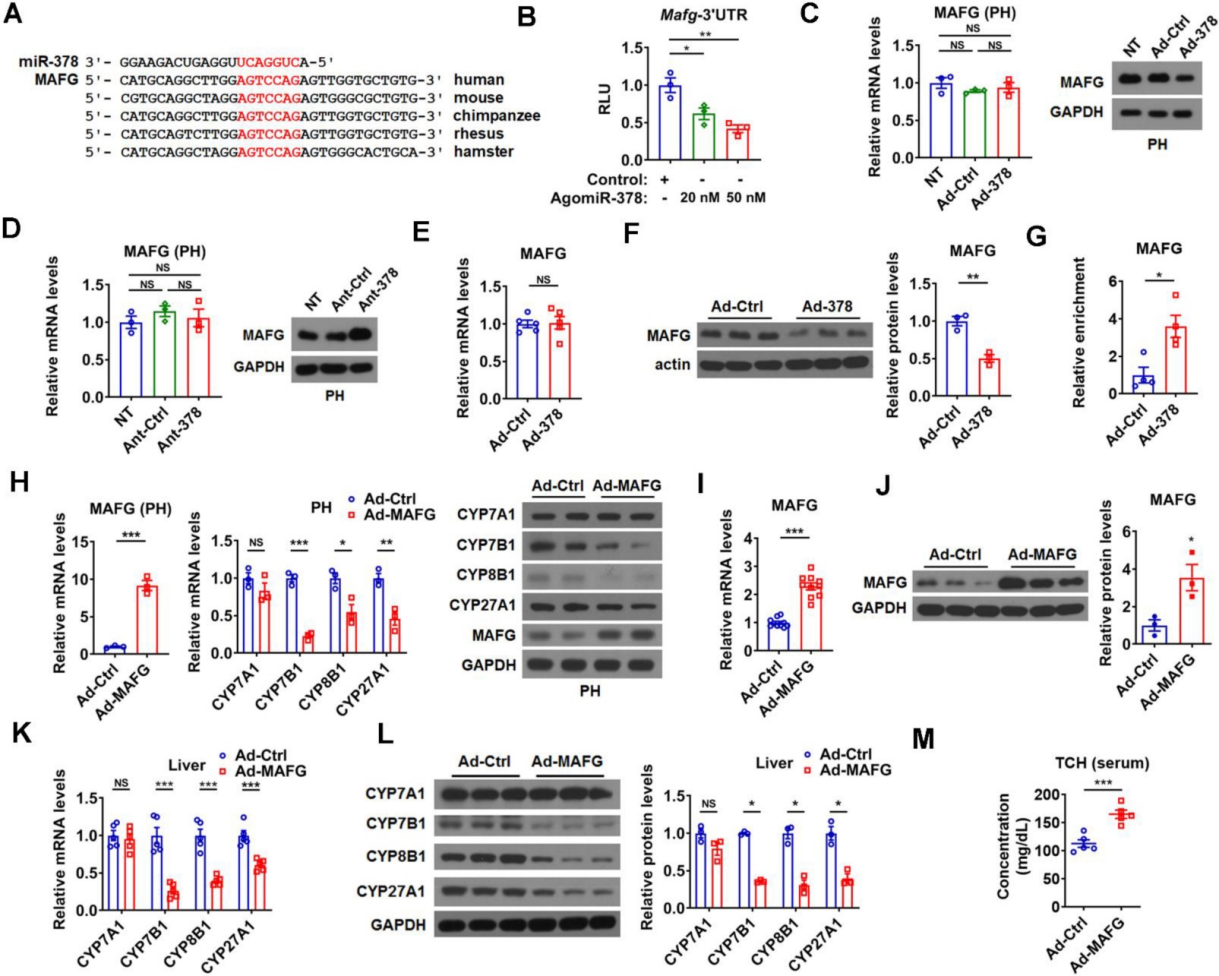
** miR-378 directly targets MAFG in the liver of mice.** (A) Sequence alignment of miR-378 and the 3'UTR of MAFG from various species. (B) Relative luciferase activity of the reporter containing the 3'UTR of MAFG in the presence of increasing amounts of AgomiR-378 in HEK293T cells (n = 3). (C) Relative mRNA levels (n = 3) and western blot analysis of MAFG in the primary murine hepatocytes (PHs) without any treatment (NT) or infected with Ad-Ctrl or Ad-378. (D) Relative mRNA levels (n = 3) and western blot of MAFG in the PHs without any treatment (NT) or transfected with Ant-Ctrl or Ant-378. (E and F) Relative mRNA levels (E, n = 5), western blot and densitometry analysis (F) of MAFG in the liver of mice infected with Ad-378. (G) Relative levels of endogenous MAFG transcripts in Ago-bound miRNA/mRNA complexes from the liver of mice infected with Ad-378 (n = 4). (H) Relative mRNA levels of MAFG and key enzymes of BA synthetic pathway (n = 3) and western blot analysis of MAFG and these key enzymes in the primary murine hepatocytes (PHs) infected with Ad-MAFG. (I and J) Relative mRNA levels (I, n = 10), western blot and densitometry analysis (J) of MAFG in the liver of mice infected with Ad-MAFG. (K and L) Relative mRNA levels (n = 5), western blot and densitometry analysis (L) of key enzymes of BA synthetic pathway in the liver of mice infected with Ad-MAFG. (M) Serum total cholesterol (TCH) levels in mice infected with Ad-MAFG (n = 5). Means ± SEM are shown. *p < 0.05; **p < 0.01; ***p < 0.001; NS, not significant.

To further understand the molecular mechanism underlying the regulation of BA metabolism by hepatic miR-378, we searched putative miR-378 target genes, focusing specifically on genes that could act as negative regulators of the intrahepatic conversion of cholesterol to BAs. We employed DAVID bioinformatics resources to perform GO enrichment analysis for the 416 putative miR-378 targets predicted by either TargetScan or miRDB and found only one gene, AKR1D1, has been implicated in BA metabolism. Given that miR-378 is a positive regulator of BA synthetic pathway and AKR1D1 is an enzyme in BA synthetic pathway, we speculated that AKR1D1 could not be involved in the positive regulation of BA synthesis by miR-378 33, 34. Thus, we decided to do literature searches for the other predicted miR-378 targets. Fortunately, we found that MAFG, a putative target gene of miR-378 predicted by both TargetScan and miRDB, was recently identified as a transcriptional repressor of BA synthetic genes 24. According to the analysis by TargetScan, *Mafg* contains a highly conserved putative miR-378 binding site in its 3' untranslated region (UTR) (Figure 3A). Moreover, we found that miR-378 overexpression repressed the luciferase activity of the reporter containing the 3'UTR of *Mafg* (MAFG-3'UTR) with the putative miR-378 binding site in a dose-dependent manner, but had no effect on the other two reporters containing mutated MAFG-3'UTR (Figure 3B and Figure S3A). Notably, the protein levels but not the mRNA levels of MAFG were decreased by Ad-378 infection in primary murine hepatocytes (Figure 3C). Accordingly, Ad-378 administration reduced the protein levels of MAFG in HepG2 cells (Figure S3B). Furthermore, AgomiR-378 treatment decreased the MAFG protein levels, while AgomiR-378* administration had no effect on MAFG expression in primary murine hepatocytes (Figure S3C-E), suggesting that miR-378 but not miR-378* contributes to the suppressive effect of Ad-378 on MAFG protein levels. Consistently, the protein levels but not the mRNA levels of MAFG were increased by Ant-378 treatment in primary murine hepatocytes (Figure 3D). In agreement with these *in vitro* data, MAFG protein levels but not mRNA levels were decreased in the liver of mice infected with Ad-378 (Figure 3E-F). Additionally, the transcripts of endogenous *Mafg* and other known and potential miR-378 targets were all enriched in Ago-bound miRNA/mRNA complexes after hepatic miR-378 overexpression (Figure 3G and Figure S3F), suggesting that miR-378 guides Ago-containing microRNA-induced silencing complex (miRISC) to MAFG transcripts. Collectively, our results suggest that MAFG is a direct target gene of miR-378.

In agreement with the previous report 24, adenovirus-mediated overexpression of MAFG (Ad-MAFG) could inhibit the expression of CYP7B1, CYP8B1, and CYP27A1, but not CYP7A1 in primary murine hepatocytes (Figure 3H). Consistently, Ad-MAFG infection decreased the luciferase activities of the reporters containing *Cyp7b1*, *Cyp8b1*, and *Cyp27a1* promoters in hepatocytes, respectively (Figure S3G). Accordingly, the occupancy of MAFG at the proximal promoter regions of these three genes could be detected by ChIP analysis in hepatocytes (Figure S3H-I), further supporting the notion that MAFG is a direct transcriptional repressor of CYP7B1, CYP8B1, and CYP27A1. Importantly, although MAFG has been implicated in the regulation of BA metabolism, whether it affects cholesterol homeostasis is not known 24. Interestingly, we found that mice infected with Ad-MAFG exhibited increased liver-specific overexpression of MAFG, decreased expression of hepatic CYP7B1, CYP8B1, and CYP27A1, and increased serum cholesterol levels (Figure 3I-M and Figure S3J-L), indicating that hepatic MAFG is not only a key regulator of BA synthesis but also a potent modulator of cholesterol homeostasis.

### MAFG mediates the effect of hepatic miR-378 on BA and cholesterol metabolism

**Figure 4:**
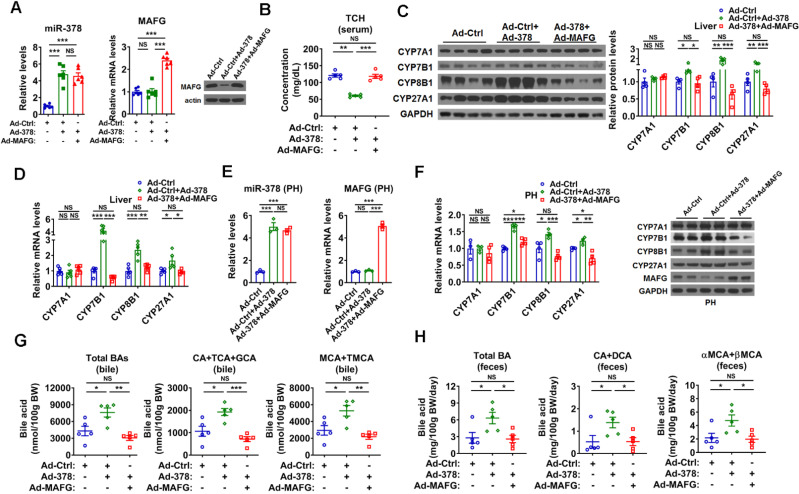
** Restoration of MAFG in the liver abolishes the effect miR-378 on cholesterol and BA metabolism.** (A) Relative levels of miR-378 and MAFG mRNA (left, n = 6) and western blot analysis of MAFG (right) in the liver of mice infected with Ad-378 and Ad-MAFG as indicated. (B) Serum total cholesterol (TCH) levels of mice infected with Ad-378 and Ad-MAFG as indicated (n = 5). (C) Western blot and densitometry analysis of key enzymes of BA synthetic pathway in the liver of mice infected with Ad-378 and Ad-MAFG as indicated. (D) Relative mRNA levels key enzymes of BA synthetic pathway in the liver of mice infected with Ad-378 and Ad-MAFG as indicated (n = 5). (E and F) Relative levels of miR-378 and MAFG mRNA (E, n = 3), relative mRNA levels and western blot analysis of key enzymes of BA synthetic pathway (F) in the primary murine hepatocytes (PHs) infected with Ad-378 and Ad-MAFG as indicated. (G and H) The levels of total and major BAs in the bile (G, n = 5) and feces (H, n = 5) of mice infected with Ad-378 and Ad-MAFG as indicated. Means ± SEM are shown. *p < 0.05; **p < 0.01; ***p < 0.001; NS, not significant.

To test whether MAFG is the primary target gene responsible for the effect of miR-378 on BA and cholesterol metabolism, we restored the protein levels of MAFG back to a normal range in the liver of Ad-378-infected mice by co-infection with Ad-MAFG, which failed to be repressed by miR-378 due to the absence of 3'UTR (Figure 4A). We found that the restoration of MAFG expression could not only abolish the effect of Ad-378 on the total serum cholesterol levels but also attenuate the effect of Ad-378 on the expression of CYP7B1, CYP8B1, and CYP27A1 in the liver of mice (Figure 4B-D and Figure S4A). Moreover, the effect of the restoration of MAFG expression on CYP7B1, CYP8B1, and CYP27A1 expression could also be observed in primary murine hepatocytes infected with Ad-378 (Figure 4E-F). Consistently, the restoration of MAFG expression in the liver of mice was capable of diminishing the effect of Ad-378 on the levels of total and major BAs in the bile and feces (Figure 4G-H). Together, these data suggest that the downregulation of MAFG is required for the action of miR-378 on BA and cholesterol homeostasis.

**Figure 5:**
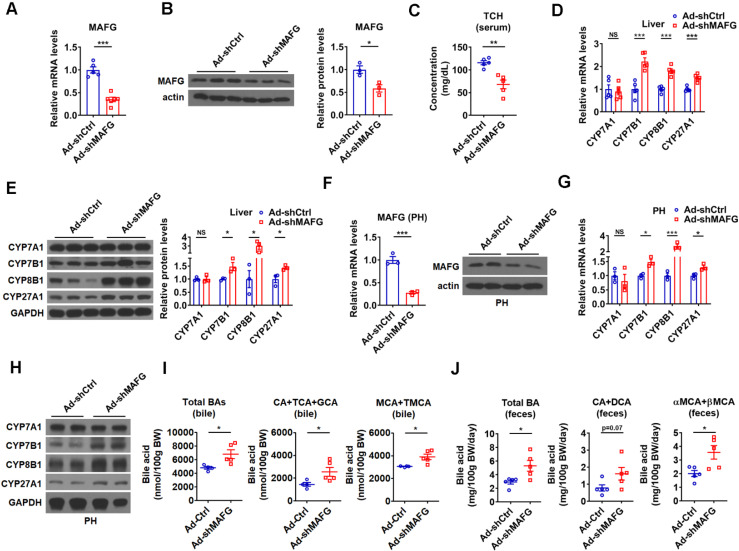
** Knockdown of hepatic MAFG enhances BA synthesis and excretion and lowers the serum total cholesterol levels.** (A and B) Relative mRNA levels (n = 5-6), western blot and densitometry analysis of MAFG in liver of mice infected with Ad-shMAFG. (C) Serum total cholesterol (TCH) levels of mice infected with Ad-shMAFG (n = 5). (D and E) Relative mRNA levels (D, n = 5), western blot and densitometry analysis (E) of key enzymes of BA synthetic pathway in the liver of mice infected with Ad-shMAFG. (F) Relative mRNA levels (n = 3) and western blot analysis of MAFG in the primary murine hepatocytes (PHs) infected with Ad-shMAFG. (G and H) Relative mRNA levels (G, n = 3) and western blot analysis (H) of key enzymes of BA synthetic pathway in the PHs infected with Ad-shMAFG. (I and J) The levels of total and major BAs in the bile (I, n = 4-5) and feces (J, n = 5) of mice infected with Ad-shMAFG as indicated. Means ± SEM are shown. *p < 0.05; **p < 0.01; ***p < 0.001; NS, not significant.

To further support the notion that MAFG mediates the effect of hepatic miR-378 on BA and cholesterol metabolism, adenovirus-mediated MAFG short hairpin RNA (shRNA) was employed to knock down MAFG. As expected, adenoviral MAFG shRNA (Ad-shMAFG) significantly and specifically decreased both MAFG mRNA and protein levels in the mouse liver (Figure 5A-B and Figure S5A). Meanwhile, the total serum cholesterol levels were significantly reduced in mice infected with Ad-shMAFG (Figure 5C). Furthermore, downregulation of MAFG in the liver of mice led to an increase in the expression of CYP7B1, CYP8B1, and CYP27A1, but had no effect on the CYP7A1 expression (Figure 5D-E). Similar observation was obtained in primary murine hepatocytes either infected with Ad-shMAFG (Figure 5F-H) or transfected with five shRNA plasmids targeting different regions of MAFG (Figure S5B-C).

Accordingly, Ad-shMAFG infection increased the levels of total BAs and major CAs and MCAs in the bile of gallbladders (Figure 5I). Notably, the percentage of CAs was tended to be slightly increased, while the percentage of MCAs was tended to be slightly decreased in the bile after MAFG knockdown; however, these changes did not reach statistical significance (Figure S5D). Importantly, the levels of total and major fecal BAs were correspondingly increased in the feces of mice after Ad-shMAFG infection (Figure 5J). Collectively, the above findings that knockdown of MAFG resulted in a phenotype similar to that observed in mice infected with Ad-378 further support the notion that MAFG mediates the regulatory effect of hepatic miR-378 on BA and cholesterol homeostasis.

### Moderate overexpression of hepatic miR-378 is sufficient to modulate BA and cholesterol metabolism

**Figure 6:**
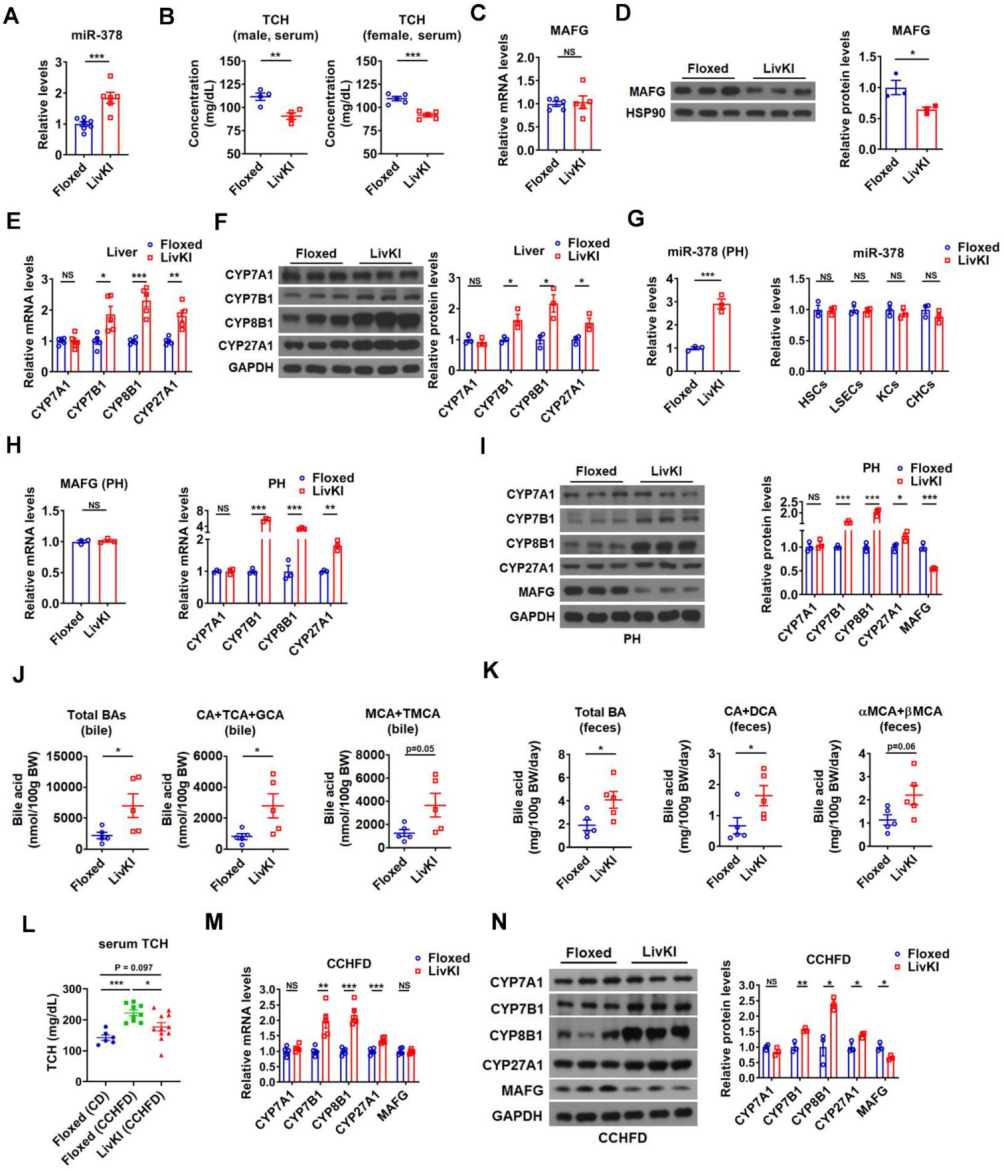
** Moderate overexpression of hepatic miR-378 in mice decreases serum cholesterol levels and enhances BA synthesis and excretion.** (A) Relative levels of miR-378 in the liver of LivKI mice (n = 6-7). (B) Serum total cholesterol (TCH) levels of male (n = 4) and female (n = 5) LivKI mice. (C and D) Relative mRNA levels (C, n = 5), western blot and densitometry analysis (D) of MAFG in the liver of LivKI mice. (E and F) Relative mRNA levels (E, n = 5), western blot and densitometry analysis (F) of key enzymes of BA synthetic pathway in the liver of LivKI mice. (G) Relative levels of miR-378 in the primary murine hepatocytes (PHs), cholangiocytes (CHCs), hepatic stellate cells (HSCs), liver sinusoidal endothelial cells (LSEcs), and Kupffer cells (KCs) from LivKI mice (n = 3). (H) Relative mRNA levels of MAFG and key enzymes of BA synthetic pathway in the primary murine hepatocytes (PHs) of LivKI mice (n = 3). (I) Western blot and densitometry analysis of key enzymes of BA synthetic pathway in the PHs of LivKI mice. (J and K) The levels of total and major BAs in the bile (J, n = 5) and feces (K, n = 5) of LivKI mice. (L) Serum TCH levels of Floxed and LivKI mice fed with control diet (CD) or cholesterol-containing high-fat diet (CCHFD) (n = 6-11). (M and N) Relative mRNA levels (M, n = 5), western blot and densitometry analysis of MAFG and key enzymes of BA synthetic pathway in the liver of LivKI mice fed with CCHFD. Means ± SEM are shown. *p < 0.05; **p < 0.01; ***p < 0.001; NS, not significant.

To investigate the effect of miR-378 on BA and cholesterol metabolism under a more physiologically relevant condition, we generated a liver-specific miR-378/378* transgenic mouse model (LivKI mice) (Figure 6A and Figure 6SA-C). Notably, the extent of the elevation of hepatic miR-378 levels in LivKI mice was comparable to that seen under fasting conditions (Figure 6A) 26. Interestingly, moderate and specific overexpression of hepatic miR-378 led to a significant reduction of total serum cholesterol levels without changing the hepatic cholesterol levels in male LivKI mice compared to Floxed mice (Figure 6B and Figure S6D). A decrease in total serum cholesterol levels could also be observed in female LivKI mice (Figure 6B). Moreover, the MAFG protein levels but not mRNA levels were reduced in the liver of LivKI mice (Figure 6C-D). Accordingly, the expression of CYP7B1, CYP8B1, and CYP27A1, but not CYP7A1 was increased in the liver of LivKI mice (Figure 6E-F). Similar results were obtained in primary murine hepatocytes derived from LivKI and Floxed mice (Figure 6G-I and Figure S6E). To be noted, the mRNA levels of genes related to cholesterol uptake (LDLR, PCSK9, SR-BI) and cholesterol* de novo* synthesis (HMGCR, SREBP2) were not altered in the liver of LivKI mice (Figure S6F). Consistently, the levels of total and major BAs were significantly or tend to be increased in the bile and feces, while the percentages of major BAs were not altered in the bile of LivKI mice (Figure 6J-K and Figure S6G). These data suggest that moderate overexpression of hepatic miR-378 is sufficient to lower serum cholesterol levels by promoting hepatic BA synthesis and BA excretion. More interestingly, as compared to Floxed mice, the LivKI mice were resistant to diet-induced hypercholesterolemia (Figure 6L). As expected, the mRNA and protein levels of CYP7B1, CYP8B1 and CYP27A1 were higher, while the protein levels of MAFG were lower in LivKI mice than those in Floxed mice under CCHFD diet (Figure 6M-N), suggesting that liver-specific overexpression of miR-378 could prevent mice from diet-induced hypercholesterolemia by promoting the BA synthesis and hepatic miR-378 might serve as a potential target for the treatment of hypercholesterolemia.

### Loss of miR-378/378* leads to defects in BA and cholesterol homeostasis

**Figure 7:**
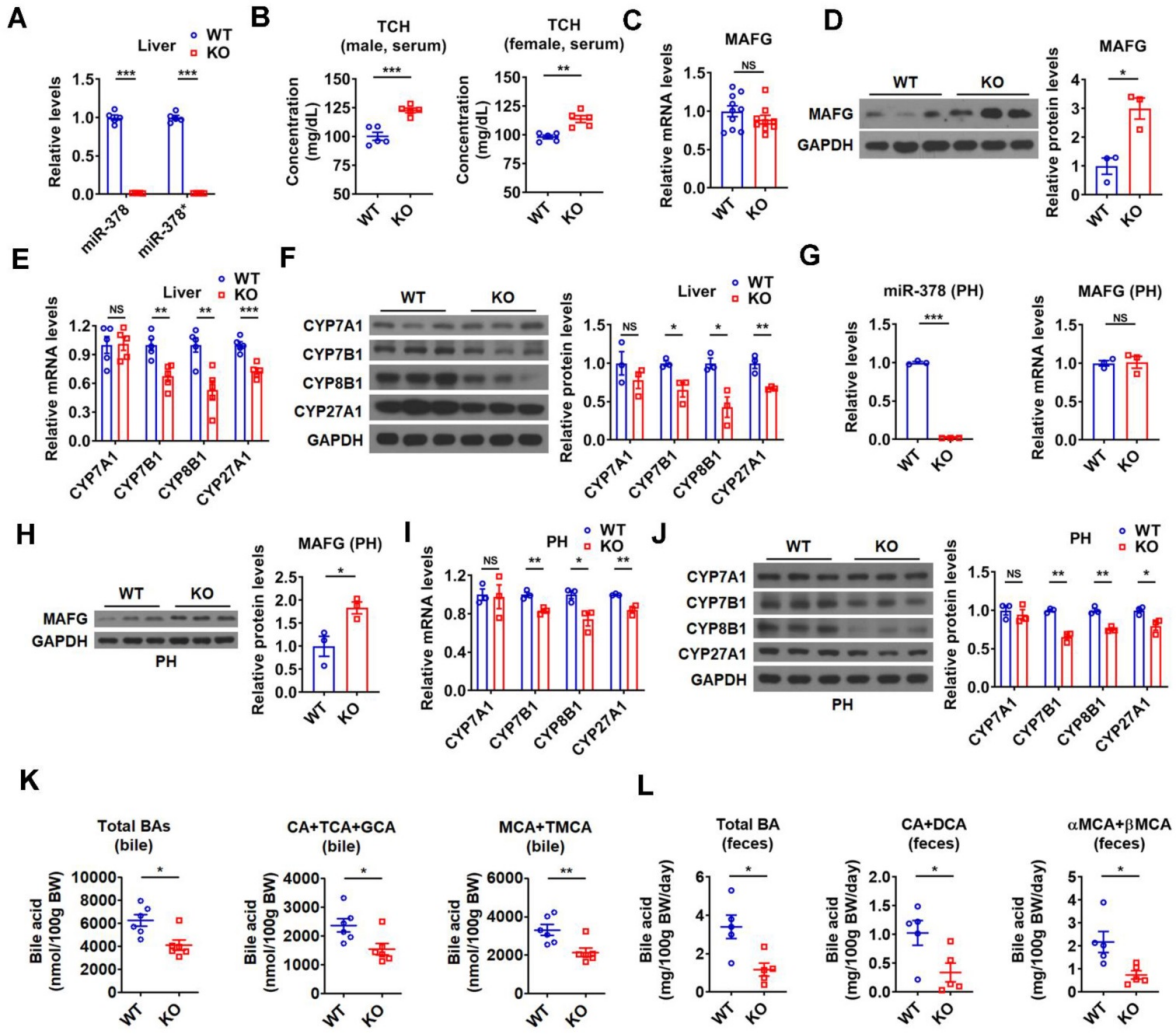
** Mice lacking miR-378 have defects in cholesterol and BA homeostasis.** (A) Relative levels of miR-378 and miR-378* in the liver of WT and KO mice (n = 5). (B) Serum total cholesterol (TCH) levels of WT and KO mice (n = 5). (C) Relative mRNA levels (n = 9-10) of MAFG in the liver of WT and KO mice. (D) Western blot and densitometry analysis of MAFG in the liver of WT and KO mice. (E and F) Relative mRNA levels (E, n = 5), western blot and densitometry analysis (F) of key enzymes of BA synthetic pathway in the liver of WT and KO mice as indicated. (G) Relative levels of miR-378 and MAFG mRNA (n = 3) in the primary murine hepatocytes (PHs) of WT and KO mice. (H) Western blot and densitometry analysis of MAFG in the primary murine hepatocytes (PHs) of WT and KO mice. (I and J) Relative mRNA levels (I, n = 3), western blot and densitometry analysis (J) of key enzymes of BA synthetic pathway in the primary murine hepatocytes (PHs) of WT and KO mice. (K and L) The levels of total and major BAs in the bile (K, n = 6) and feces (L, n = 5) of WT and KO mice as indicated. Means ± SEM are shown.*p < 0.05; **p < 0.01; ***p < 0.001; NS, not significant.

To further investigate the regulatory role of endogenous miR-378 in BA and cholesterol homeostasis, we took advantage of global miR-378/378* knockout (KO) mice developed previously (Figure 7A) 26. In line with above results, an increase in serum cholesterol levels was observed in the male KO mice, while hepatic cholesterol levels were not altered (Figure 7B and Figure S7A). An elevation of serum cholesterol levels could be observed in female KO mice (Figure 7B). Consistent with our above findings, the protein levels but not the mRNA levels of MAFG were increased in the liver of KO mice as compared to wild-type (WT) mice (Figure 7C-D). Moreover, the expression of CYP7B1, CYP8B1, and CYP27A1 was decreased, while the expression of CYP7A1 was not altered in the liver of KO mice (Figure 7E-F). As expected, the expression of genes related to cholesterol uptake (LDLR, PCSK9, SR-BI) and cholesterol *de novo* synthesis (HMGCR, SREBP2) was not changed in the liver of KO mice (Figure S7B). The effect of miR-378 deficiency on the expression of MAFG, CYP7B1, CYP8B1, and CYP27A1 was also observed in primary murine hepatocytes derived from KO mice (Figure 7G-J). Accordingly, the levels of total and major BAs were all decreased in the bile and feces of KO mice (Figure 7K-L), suggesting a reduction in hepatic BA synthesis and a corresponding decrease in BA excretion. Additionally, no significant changes in the BA composition were observed in the bile of KO mice (Figure S7C). Collectively, these results suggest that miR-378 is required for maintaining normal BA and cholesterol homeostasis.

Since results obtained in the murine hepatocytes or mouse models may not be extrapolated to humans, to test whether the regulation of MAFG and its target genes by miR-378 could be observed in human cells, we employed human hepatocyte-like cells (hHLCs) derived from human pluripotent stem cells (hPSCs) as described before 35. We found that, similar to those observed in murine cells and mouse models, miR-378 overexpression only altered the protein levels but not the mRNA levels of MAFG in hHLCs (Figure S7D-E). Moreover, overexpression of miR-378 increased the expression of CYP7B1, CYP8B1, and CYP27A1, but not CYP7A1 in hHLCs (Figure 7D-E). These observations implicate a conserved role of miR-378 in orchestrating hepatic BA synthesis.

Collectively, based on our above data, we propose that hepatic miR-378 regulates both classic and alternative BA synthetic pathways by derepressing the transcription of CYP7B1, CYP8B1 and CYP27A1 via its direct target MAFG, thereby modulating BA and cholesterol homeostasis (Figure S7F).

### miR-378 is involved in the regulation of BA and cholesterol metabolism by TH

**Figure 8:**
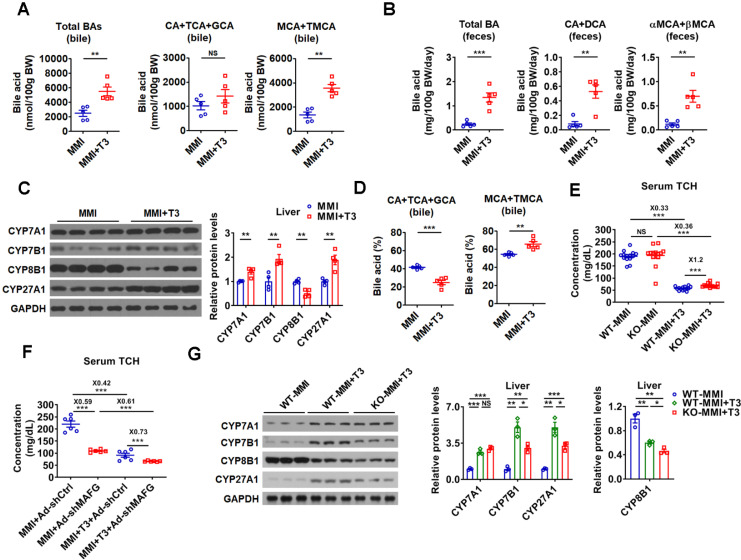
** miR-378 is involved in the regulation of BA and cholesterol metabolism by TH.** (A and B) The levels of total and major BAs in the bile (A, n = 5) and feces (B, n = 5) of hypothyroid (MMI) and hyperthyroid (MMI+T3) mice as indicated. (C) Western blot and densitometry analysis of key enzymes of BA synthetic pathway in the liver of hypothyroid (MMI) and hyperthyroid (MMI+T3) mice. (D) BA composition in the bile of hypothyroid (MMI) and hyperthyroid (MMI+T3) mice as indicated (n = 5). (E) Serum total cholesterol (TCH) levels of hypothyroid (MMI) and hyperthyroid (MMI+T3) WT and KO mice (n = 13-14). (F) Serum total TCH levels in hypo- and hyperthyroid mice infected with Ad-shMAFG or Ad-shCtrl (n = 6). 24 C57BL/6 mice were infected with Ad-shMAFG and Ad-shCtrl (12 mice in each group), respectively, and then rendered hypothyroid (MMI) for 2 weeks. After that, six hypothyroid Ad-shMAFG-infected mice and six hypothyroid Ad-shCtrl-infected mice received 5 daily T3 injections, respectively, which were killed 2 h after last T3 injection. (G) Western blot and densitometry analysis of key enzymes of BA synthetic pathway in the liver of hypothyroid (MMI) and hyperthyroid (MMI+T3) WT and KO mice as indicated. Means ± SEM are shown. *p < 0.05; **p < 0.01; ***p < 0.001; NS, not significant.

In line with current knowledge and our findings, the BA levels were higher in the bile and feces of hyperthyroid mice than those of hypothyroid mice, while no differences were observed in the serum and hepatic BA levels between hypothyroid and hyperthyroid mice (Figure 8A-B and Figure S8A). In agreement with these data, the levels of total and major fecal BAs were all decreased in hypothyroid patients and increased in hyperthyroid patients as compared to normal human subjects, which were inversely correlated with serum cholesterol levels in these subjects (Figure S8B). Additionally, in line with our knowledge that the key enzymes in BA synthetic pathway were differentially regulated by T3 treatment, the expression of CYP7A1, CYP7B1, and CYP27A1 was positively regulated by T3, while the CYP8B1 expression was negatively regulated by T3 (Figure 8C and Figure S8C). Consistent with the positive regulation of CYP7A1 by TH, the serum C4 levels were elevated by T3 treatment (Figure S8D). In agreement with the negative regulation of CYP8B1 by TH, the percentage of CAs was decreased, while the percentage of MCAs was increased in the bile after T3 administration (Figure 8D).

As hepatic miR-378 was initially identified as a TH-responsive miRNA (Figure 1), we explored the role of miR-378 in TH-regulated cholesterol and BA metabolism. Interestingly, the serum cholesterol levels were 20% higher after T3 treatment in hypothyroid KO mice as compared to hypothyroid WT mice (Figure 8E), suggesting that KO mice were less sensitive to T3 treatment. Based on these data, we propose that the cholesterol-lowing effect of T3 is partially dependent on miR-378. Additionally, in agreement with the notion that MAFG acts as a downstream effector of miR-378, knockdown of hepatic MAFG by Ad-shMAFG retained the cholesterol-lowing capacity in hypothyroid mice with reduced basal hepatic miR-378 levels (Figure 8F and Figure 1B). Also as expected, mice infected with Ad-shMAFG were less sensitive to T3 treatment (Figure 8F), indicating MAFG is involved in the regulation of serum cholesterol levels by TH. Collectively, based on these data, we propose that the cholesterol-lowing effect of T3 is partially dependent on miR-378 and its target MAFG in the liver of mice.

In addition, consistent with the regulatory role of miR-378 discovered in this study, the ability of T3 to enhance the expression of CYP7A1 was retained, while the capacity of T3 to stimulate the expression of CYP7B1 and CYP27A1 was attenuated in hypothyroid KO mice as compared to hypothyroid WT mice (Figure 8G). Furthermore, the suppressive effect of T3 on CYP8B1 expression was more potent in hypothyroid KO mice as compared to hypothyroid WT mice (Figure 8G). Based on our findings, we also speculate that T3 and T3-responsive miR-378 might form different feed-forward loops to fine-tune the expression of CYP7B1, CYP8B1, and CYP27A1, thereby conferring robust and precise controls on both classic and alternative BA synthetic pathways (Figure S8E).

Taken together, our study defines hepatic miR-378 as an important modulator of BA and cholesterol homeostasis. Hepatic miR-378 reduces the serum cholesterol levels through promoting the hepatic BA synthesis and BA excretion by targeting MAFG, a transcriptional repressor of hepatic BA synthetic genes. Our findings also suggest that miR-378 is positively regulated by TH and involved in the regulation of BA and cholesterol metabolism by TH.

## Discussion

High plasma cholesterol levels can cause atherosclerosis, which increase the risk of chest pain, heart attack and stroke. Great efforts have been made to seek novel therapies to optimize plasma cholesterol levels. Treatments that enhance the hepatic BA synthetic pathway, a primary route to eliminate excess cholesterol from the body, should be of particular value. An improved understanding of the regulation of primary BA synthesis in the liver may help to identify novel targets or strategies to lower the levels of cholesterol and retard the progression of atherosclerosis. TH signalling pathways have been targeted as potential therapeutic avenues to lower serum cholesterol levels, partially due to the fact that TH can stimulate the intrahepatic conversion of cholesterol into BAs, thereby enhancing the cholesterol elimination. Identifying novel modulators involved in the regulation of hepatic BA synthesis by TH will not only help to understand the TH signalling pathways but also help to find novel pharmacological approaches to treat hypercholesterolemia.

In this study, we performed miRNA microarray analysis to identify hepatic TH-responsive miRNAs that may be involved in the regulation of cholesterol metabolism by TH. Fold enrichment analysis of the miRNA microarray data revealed that miRNAs ranked from 1^st^ to 4^th^ among the differentially expressed miRNAs all belong to let-7 family, which made miR-378 the 2^nd^-ranked miRNA. Further studies suggest that TH-responsive miR-378 exerts favorable cholesterol-lowering effects through suppressing its direct target MAFG, which is a transcriptional repressor of BA synthetic genes, thereby derepressing the expression of key enzymes (CYP7B1, CYP8B1, and CYP27A1) of BA synthetic pathway. Indeed, we do not intend to exclude the possibility that other differentially expressed miRNAs including let-7 family members also can regulate cholesterol metabolism and mediate the cholesterol-lowering effect of TH. It is worth noting that loss of miR-378/378* could not fully abolish the cholesterol-lowering effect of TH in hypothyroid mice, suggesting that other miRNAs or factors might also be involved in the regulation of cholesterol homeostasis by TH. Nevertheless, given that either overexpression of hepatic miR-378 or downregulation of hepatic MAFG could markedly decrease serum cholesterol levels, we propose that hepatic miR-378 and its target MAFG may serve as potential therapeutic targets.

Two approaches were usually employed to exam the hepatic BA synthesis. One way is to determine the BA levels enzymatically in different BA pools by using hydroxysteroid dehydrogenase, which likely has selectivity or specificity for different bile salt species. The other way is to measure the serum C4 levels, which closely reflects CYP7A1 activity and classic BA synthetic pathway but not alternative BA synthetic pathway. In this study, we determined the levels of bile salt species directly by using UFLC-Triple-time of flight/MS to clarify the effect of miR-378 on both classic and alternative BA synthetic pathways. We found that both the levels of CAs and MCAs were significantly increased, while the percentage of CAs and MCAs were not changed in the bile after Ad-378 administration. Given that alternative BA synthetic pathway predominantly produces MCAs, while classic BA synthetic pathway generates both MCAs and CAs in mice; our data suggest that both classic and alternative BA synthetic pathways were regulated by miR-378 (Figure S6H). These data are also consistent with our finding that miR-378 regulates the expression of CYP7B1, CYP8B1, and CYP27A, but not CYP7A1.

CYP8B1 catalyzes 12α-hydroxylation of C4 for CA synthesis. Without 12α-hydroxylation, C4 will be converted to 5β-cholestan-3α,7α-diol for CDCA synthesis 36. In this study, after the overexpression or knockdown of miR-378, the expression of CYP8B1 was significantly changed (Figure 2, 6, 7), which would affect the conversion of C4 to CA or CDCA 5. However, we could not observe obvious changes in BA composition after the expression of miR-378 was altered (Figure S2F, S6G, S7C). As the expression of CYP27A1 and CYP7B1 in the alternative pathway was also regulated by miR-378, we speculate that the regulation of alternative pathway by miR-378 through CYP27A1 and CYP7B1 might decrease the CYP8B1-caused changes in the ratio of CA and CDCA/MCA. Additionally, given that the BA pool cycles between the liver and the intestine (enterohepatic circulation) and BA composition could be affected by many other factors, including gut microbiota 32, 37, we also speculate that other factors or mechanisms might also be involved to compromise the effect of CYP8B1 on BA composition when hepatic miR-378 expression altered.

In this study, we observed that hepatic miR-378 is able to regulate serum cholesterol levels without affecting the expression of CYP7A1, the rate limiting enzyme of classic BA synthetic pathway. In agreement with our findings, it has been reported that serum cholesterol levels were increased and fecal BA excretion was decreased in CYP27A1 heterozygous knockout male mice 38. Furthermore, it has been proposed that cold-induced conversion of cholesterol to BAs is dependent on hepatic CYP7B1 induction 39. These two studies imply that the upregulation of CYP27A1 and CYP7B1 might be sufficient to enhance hepatic BAs synthesis, thereby decreasing serum cholesterol levels. Thus, our study here not only further emphasizes the importance of CYP27A1 and CYP7B1 in maintaining normal cholesterol homeostasis, but also indicates that CYP27A1 and CYP7B1 might serve as potential therapeutic targets for lowering serum cholesterol levels.

miR-378 plays important roles in metabolic regulation. MiR-378 transgenic mice display increased energy expenditure 40, while mice lacking miR-378/378* are resistant to obesity and exhibit enhanced mitochondrial fatty acid metabolism 41. Here, our gene ontology analysis revealed that the hepatic genes regulated by miR-378 were clustered and enriched in many metabolic pathways (Figure 2A). Notably, we previously demonstrated that miR-378 is positively regulated by fasting and it regulates insulin signalling 26 and autophagy in liver and muscle, respectively 27. In this study, we discovered an essential role of hepatic miR-378 in the regulation of hepatic BA and cholesterol homeostasis. It has been suggested that both hepatic BA synthesis and gluconeogenesis are regulated by insulin signalling and coordinated by the fasting-to-fed cycle, while BA pool size also alters under diabetic conditions 5. Thus, whether miR-378 plays a role in the regulation of hepatic BA synthesis upon fasting or in diabetes needs further investigation.

Recently, Shelly C. Lu's lab reported that hepatic MAFG expression is induced in chronic cholestasis, which contributes to the cholestatic liver injury. Moreover, knockdown of MAFG could protect against cholestatic liver injury 42, 43. In this study, we demonstrate that hepatic MAFG is a direct target of miR-378. Given that several miRNA-based therapeutics have advanced into clinical testing, it would be interesting to test whether overexpression of hepatic miR-378 could improve cholestatic liver disease by downregulating MAFG in the future. It is also worth noting that NRF1, a guardian of cholesterol homeostasis 44, has been shown to heterodimerize with MAFG and form a negative feedback loop with miR-378 in the pathogenesis of hepatosteatosis 45. Moreover, NRF2-MAFG heterodimers have been implicated in regulating genes related to metabolic processes 46. As PGC-1β, the miR-378 host gene, also regulates hepatic lipid metabolism and energy homeostasis, how PGC-1β/miR-378 and NRF1/2-MAFG heterodimers participate in the regulatory network to coordinate lipid metabolism requires further investigation.

In summary, our results establish hepatic T3-responsive miR-378 as a novel regulator of cholesterol homeostasis and hepatic BA synthesis. Our results also demonstrate that MAFG might be the primary target that mediates the effect of hepatic miR-378 on BA and cholesterol metabolism, while miR-378 and MAFG could serve as therapeutic targets for the treatment of hypercholesterolemia.

## Materials and Methods

### Animal study

Wild-type C57BL/6 mice or transgenic mice on the C57BL/6 background aged about 2-4 months were used in this study. Age-matched mice or mice from same litters were randomly assigned to each group. Male mice were used, unless otherwise mentioned. All *in vivo* experiments described here were in accordance with institutional guidelines for the care and use of animals. All animal protocols were approved by the Animal Care Committee (2015-AN-12, 2016-AN-1, SIBS-2019-YH-1). A conditional miR-378/miR-378* transgenic mouse line was generated, allowing exogenous miR-378/miR-378* expression in a Cre-recombinase-dependent manner. Briefly, the expression cassette pA-EGFP-loxp-loxp2272-EF1a-loxp-loxp2272-miR-378/miR-378*-pA was inserted into the *Rosa26* gene locus by ES cell targeting. The initial orientation of EF1a promoter does not initiate transcription of the downstream target gene miR-378/378*. To achieve high expression of miR-378/378* driven by the reversed *Ef1a* promoter in the liver of mice, these transgenic mice were mated with the Alb-Cre mice to obtain the liver-specific miR-378/378* transgenic mice (LivKI mice). Primers used for genotyping are as follows: 378P1:5'-AGG CAG GGT CTC ACT ATG TAT CTC-3'; 378P2:5'-ATG GGG GAA AAC TTG AAT GAA-3'; 378P3:5'-CTC CCC CGT GCC TTC CTT GAC-3'. MiR-378/378* knockout (KO) mice were developed in our lab as described before 26. *Thrb* knockout mice were kindly gifted from Douglas Forrest (NIDDK) 30.

Male C57BL/6 or miR-378 KO mice were rendered hypothyroid by the addition of 0.1% methimazole (MMI) and 1% NaClO_4_ in their drinking water for two weeks. Hyperthyroidism was induced in some of these hypothyroid mice by intraperitoneal injection of T3 every day (5 µg/20 g body weight) for 5 days. These hyperthyroid mice were sacrificed 2 h after last T3 injection. Hypercholesterolemia was induced in male Floxed and miR-378 LivKI mice by feeding them a high-fat rodent diet with 1.25% cholesterol (Research Diets, D12108C), which are commonly used to establish a hypercholesterolemia model 47, for 3 months. Adenoviruses were used to manipulate the hepatic expression of miR-378 or MAFG in mice. Each C57BL/6 mouse received recombinant adenovirus (0.1 ~ 1x10^9 PFU) through tail vein injection, and then sacrificed two weeks after injection. To specifically knock down the expression of miR-378 in the liver of mice, C57BL/6 mice were intraperitoneally injected with miRNA miR-Down^TM^ antagomir of miR-378 at a low dose (10 nmol/25 g body weight), and then sacrificed after four days. Regarding the rescue experiments, C57BL/6 mice aged about 8 weeks were infected with Ad-Ctrl (adenovirus with an empty vector), co-infected with Ad-378 and Ad-Ctrl or co-infected with Ad-378 and Ad-MAFG as indicated. In this experiment, equal amount of Ad-378 and Ad-MAFG was used, while Ad-Ctrl was used to balance the total amount of infected adenoviruses. To examine the role of MAFG in T3-regulated cholesterol homeostasis, 24 C57BL/6 mice were infected with Ad-shMAFG and Ad-shCtrl (12 mice in each group), respectively, and then rendered hypothyroid (MMI) for 2 weeks. After that, six hypothyroid Ad-shMAFG-infected mice and six hypothyroid Ad-shCtrl-infected mice received 5 daily T3 injections, respectively, which were killed 2 h after last T3 injection. Regarding the measurement of food intake and feces output, mice were individually housed for 3 days in cages. Food consumption was monitored and feces were collected, dried, and weighed every 24 h. The amount of food intake and feces output was normalized to the body weight of mice. The sera and feces were collected from hypothyroid and hyperthyroid patients after obtaining their written informed consent, which was approved by the Ethics Committee of Zhongshan Hospital, Fudan University.

### Plasmids, adenovirus, and synthetic RNA

Adenoviruses recombination and administration were performed as described before 26. Briefly, the BLOCKiT Adenoviral RNAi Expression System (Invitrogen) and 293A cells were employed to generate the recombinant adenoviruses for the knockdown of MAFG expression, while the AdEasy Adenoviral Vector System (Stratagene) and 293A cells were used to prepare the recombinant adenoviruses for miR-378/378* and MAFG overexpression. Adenovirus with an empty vector (Ad-Ctrl or Ad-shCtrl) was used as control. To downregulate MAFG expression level in cells, several shRNAs for *Mafg* were designed and cloned into pENTR™/U6 vector. AgomiR-378 (miRNA miR-Up^TM^ agomir, ACU GGA CUU GGA GUC AGA AGG), AgomR-378* (miRNA miR-Up^TM^ agomir, CUC CUG ACU CCA GGU CCU GUG U), Ant-378 (miRNA miR-Down^TM^ antagomir, CCU UCU GAC UCC AAG UCC AGU), and scrambled controls were obtained from Genepharma. Primer sequences for plasmid construction and siRNA sequences are provided in Table S2.

### Cell culture and treatment

The isolation and culture of primary mouse hepatocytes, and the culture of HEK293T, Hepa1-6 and HEK293A cells were described previously 26. Hepatic stellate cells (HSCs) were isolated from the anesthetized mice by two-step protocol (collagenase/pronase digestion and Nycodenz gradient) 48. For cholangiocytes isolation, biliary tree was isolated by collagenase IV, DNase I, and pronase E after the two-step collagenase perfusion and removing liver envelope and shaking with HBSS buffer. Cholangiocytes were further collected by using 70 μm cell strainer. Liver sinusoidal endothelial cells (LSECs) and Kupffer cells (KCs) were isolated from the liver by using two-step isolation technique and commercially available magnetic beads specific for KCs (F4/80) and LSECs (CD146) 49. To verify mmu-miR-378-3p is T3-responsive, primary mouse hepatocytes were maintained in TH-deficient culture medium (Td medium), then 100 nM of T3 was administrated for indicated time or different doses of T3 were administrated for 24 h. Finally, RNA was extracted and the expression of mmu-miR-378 was analyzed. Human hepatocyte-like cells (hHLCs) were derived from human pluripotent stem cells (hPSCs) according to the protocol as described before 35. To study the roles of miR-378 and MAFG* in vitro*, isolated primary hepatocytes, Hepa1-6 cells and hHLCs were harvested for analysis after infection with the corresponding adenoviruses at a multiplicity of infection of 10 for 36 h. Agomir-378 or Ant-378 were transfected into primary mouse hepatocytes by using Lipofectamine 2000 at a final concentration of 20 nM if not specially pointed out. 36 h after transfection, cells were harvested for further analysis. To knock down MAFG in primary mouse hepatocytes, five shRNA plasmids targeting different regions of *Mafg* were transfected by using Lipofectamine 2000 according to the manual. 10 µg of HA-TRβ or HA-MAFG was transfected into Hepa1-6 cells cultured in 10 cm dish by using Lipofectamine 2000, cells were harvested for ChIP assay after 36 h.

### Reporter assay

Luciferase assay was performed as described before 26, 27. The 3'UTR of *Mafg* with the putative miR-378 binding site was cloned into pRL-TK plasmid. Then, this luciferase reporter plasmid and firefly luciferase plasmid pGL3 were co-transfected together with AgomiR-378 or Agomir control into HEK293T cells. Luciferase activity was measured using Dual luciferase Reporter Assay System (Promega, E1910) and normalized to respective controls 48 h after transfection. KOD-PLUS mutagenesis kit (Toyobo, SMK-101) was used to generate mutations in the 3'-UTR of *Mafg*. The binding sites for miR-378 were changed as follows: Mut1 (5'-AGU CCA G-3' to 5'-AGU CGA G-3') and Mut2 (5'-AGU CCA G-3' to 5'-AGU GGA G-3'). The promoter regions (~ - 2.5 to ~ + 0.5 kb) of mouse* Cyp7b1*,* Cyp8b1* and *Cyp27a1* were cloned into pGL3basic plasmid. The corresponding firefly luciferase reporter plasmids and renilla luciferase plasmid (pRL-TK) were co-transfected into Hepa1-6 cells infected with Ad-Ctrl or Ad-MAFG, Ad-378. Primers used for plasmid construction are shown in the Table S2.

### Quantification of mRNA and protein levels

Total RNA was isolated by using Trizol reagent from tissues or cells. 0.5 µg of RNA was reverse-transcribed into cDNA by using PrimeScript RT reagent Kit (TaKaRa, RR037A). Real-Time PCR was performed by using ABI Real-Time System (Applied Biosystems). Primers for RT-PCR are shown in the Table S2. Cells or tissues were lysed in RIPA lysis buffer (P0013B, Beyotime) supplemented with phosphatase and protease inhibitors. The lysates were subjected to immunoblotting, resolved on SDS-PAGE, transferred onto PVDF membranes and incubated with various primary antibodies overnight at 4 ℃.The next day, the membranes were incubated with the secondary antibodies for one h at room temperature, followed by measurement of the chemiluminescent signal. NIH Image J was employed for densitometry analysis. Densitometry analysis for the western blots in Figure 2F and 2G, Figure 3C, 3D, and 3H, Figure 4A and 4F, Figure 5F and 5H, and Figure S5C were provided in Table S3. Antibodies were obtained from Abcam (CYP7B1, ab136801; CYP8B1, ab191910; CYP27A1, ab126785), Santa Cruz (CYP7A1, sc-25536), Novus Biologicals (MAFG, NBP2-15019), Sigma (Tubulin, T5168-2ML; actin, A5441-100UL), and cell signaling (GAPDH, 3683; HSP90, 4877). For ELISA analysis, ~50 mg liver samples were homogenized in lysis buffer (150 mM NaCl, 10 mM HEPES PH 7.4, 0.5% Triton X-100) with antiprotease cocktail (Thermo Fisher), snap-frozen, thaw on ice three times, and cleared by centrifugation, followed by ELISA analysis using CYP7A1 (EK10945-1), CYP7B1 (EK10940-1) and CYP8B1 (EK10937) ELISA kit (Signalway Antibody).

### Microarray and RNA-seq data analysis and bioinformatics tools

Microarray analysis was performed to identify TH-responsive miRNAs. To identify hepatic miRNAs having maximal T3 responsiveness, mice were rendered hypothyroid first and then injected intraperitoneally with a single dose of T3 (5 µg/20 g body weight). These mice were killed 2 h after T3 injection. Hepatic RNA samples were labeled using the miRCURY™ Hy3™/Hy5™ Power labeling kit and hybridized on the miRCURY™ LNA Array (v.10.0). The analysis was performed by using the Axon GenePix 4000B microarray scanner and GenePix pro V6.0. Pathway analysis of miRNA microarray data was performed by using GO reverse search (mirPath v.3 from DIANA-miRPath web server) through TarBase v7.0 in a category called “cholesterol metabolic process | GO:0008203” to identify which TH-regulated miRNAs might be involved in cholesterol metabolism. Prediction of miR-378 targets was performed by using TargetScan (targetscan.org) and miRDB (mirdb.org). Gene-enrichment and functional annotation analysis was performed by using DAVID tools (david.ncifcrf.gov) for all predicted miR-378 targets to identify which predicted miR-378 targets might be involved in cholesterol metabolism.

RNA-seq was performed to identify genes regulated by miR-378. Libraries were prepared according to the Illumina TruSeq protocol and pooled for deep sequencing by using Illumina Hiseq Xten (2 x 150) platforms at the CAS-MPG Partner Institute for Computational Biology Omics Core, Shanghai, China. Raw read qualities were evaluated with FastQC (bioinformatics.babraham.ac.uk, v0.11.5). Adaptor sequences and read sequences on both ends with Phred quality scores below 30 were trimmed. Trimmed reads were then mapped with the Hisat2 algorithm (Hisat2 v2.1.0) to target sequences. Gene expression levels were quantified by the software package HTSeq (v0.6.1p1). The list of differentially expressed genes was generated by DESeq2 with P < 0.05. KEGG pathway and GO analysis were performed for differentially expressed genes. Data from microarray and RNA-seq analysis have been deposited in the GEO (GSE139961, GSE139830) and NODE database (OEP000637: OEX002057).

### Quantification of cholesterol, BA, and C4 levels in mice

Serum total cholesterol levels were determined according to the manufacturer's instructions (Wako, 294-65801). The bile samples from gallbladders were harvested after mice were fasted for 6 h, while the feces were collected over a 24-h period. BAs were extracted in 70% ethanol at 55 ℃ for 4 h, and bile salt species were analyzed by ultra-fast liquid chromatography (UFLC)-Triple-time of flight/mass spectrometer (MS) 50. Briefly, the chromatographic system consisted of an Ekspert ultra LC 100 system performed on AB SCIEX Triple TOF 5,600 MS, while the chromatographic separation was carried out on a Waters XBridge Peptide BEH C18 column. The total BA levels in the bile of gallbladders were calculated by summing the values of 19 BA species, including CA, αMCA, βMCA, UDCA, HDCA, CDCA, DCA, TCA, TαMCA, TβMCA, TUDCA, THDCA, TCDCA, TDCA, TLCA, GCA, GUDCA, GCDCA, and GDCA. The total BA levels in the feces of mice were calculated by summing the values of 20 BA species, including CA, αMCA, βMCA, DHCA, UDCA, HDCA, CDCA, DCA, LCA, TCA, TαMCA, TβMCA, TUDCA, THDCA, TCDCA, TDCA, TLCA, GCA, GCDCA, and GDCA. The serum C4 levels were measured as described before 50. In brief, 30 μl of serum or C4 standard (Avanti) were mixed with acetonitrile. The samples were vortexted and then spun to precipitate particulates. 20 μl of supernatant was separated by reverse phase on a Prominence 20 UFLCXR system (Shimadzu, Columbia, MD) with a Waters BEH C18 column (100 mm×2.1 mm, 1.7 µm particle size) and the eluate was delivered into a 5600 TripleTOF using a Duospray ion source (SCIEX, Framingham, MA) operating in enhanced mode and monitoring the transition m/z 401.3-177.1.

### ChIP and RIP analysis

ChIP assay was performed by using EZ Magna ChIP G kit (millipore, 17-409). For qPCR analysis, DNA samples were diluted and determined as described above. The PCR products were also analyzed in 3% agarose gel. Primer sequences for ChIP assay are provided in Table S2. Enrichment of the transcripts in Ago-bound miRNA/mRNA complexes was accessed by performing AGO2 pull down assay with Magna RIP Kit (millipore, 17-701). The lysate of liver (about 100 mg) of mice infected with Ad-Ctrl or Ad-378 and magnetic beads were incubated with anti-AGO2 (Abcam, ab32381) or anti-IgG (millipore) at 4 ℃ for overnight with shaking. The immunoprecipitate was incubated with proteinase K at 55 ℃ for 30 min. The RNA was purified, followed by reverse-transcribed PCR using primers targeting the 3'UTR region of the genes. The amount of transcripts in Ago-bound miRNA/mRNA complexes was measured and demonstrated using threshold cycles after weight normalization. The mean value in the samples from mice infected with Ad-Ctrl was set to 1.0. Primer sequences for RIP assay are provided in Table S2.

### Statistical Analysis

Sample size for each experiment was determined by PS Power and Sample Size Calculations (Version 3.0). All experiments in this study were performed more than twice, and representative data are shown. Data were expressed as means ± SEM. Student's t test was performed to compare the means of two experimental groups by using GraphPad Prism. A P value of less than 0.05 was considered significant. False discovery rate was calculated and used for RNA-seq data analysis to identify differentially expressed genes.

## Supplementary Material

Supplementary figures and tables.

## Abbreviations

miR-378: mmu-miR-378-3p
miR-378*: mmu-miR-378-5p
BA: bile acid
TH: thyroid hormone
TR: thyroid hormone receptor
C4: 7α-hydroxy-4-cholesten-3-one
CYP7A1: cytochrome P450, family 7, subfamily a, polypeptide 1
CYP7B1: cytochrome P450, family 7, subfamily b, polypeptide 1
CYP8B1: cytochrome P450, family 8, subfamily b, polypeptide 1
CYP27A1: cytochrome P450, family 27, subfamily a, polypeptide 1
MAFG: v-maf musculoaponeurotic fibrosarcoma oncogene family, protein G
CA: cholic acid
CDCA: Chenodeoxycholic acid
TCA: taurine-conjugated cholic acid
GCA: glycine-conjugated cholic acid
MCA: muricholic acid
TMCA: taurine-conjugated muricholic acid
DCA: deoxycholic acid
Ago: argonaute
miRISC: microRNA-induced silencing complex
ChIP: Chromatin immunoprecipitation
RIP: RNA binding protein immunoprecipitation
hHLCs: human hepatocyte-like cells
UTR: untranslated region
