# Supplementary material for: Hepatic miR-378 modulates serum cholesterol levels by regulating hepatic bile acid synthesis
Source: Theranostics. 2021 Feb 25;11(9):4363–80. doi: 10.7150/thno.53624 (PMC7977473; doi:10.7150/thno.53624)
Supplement: Supplementary file 1 — Supplementary figures and tables. [file thnov11p4363s1.pdf]

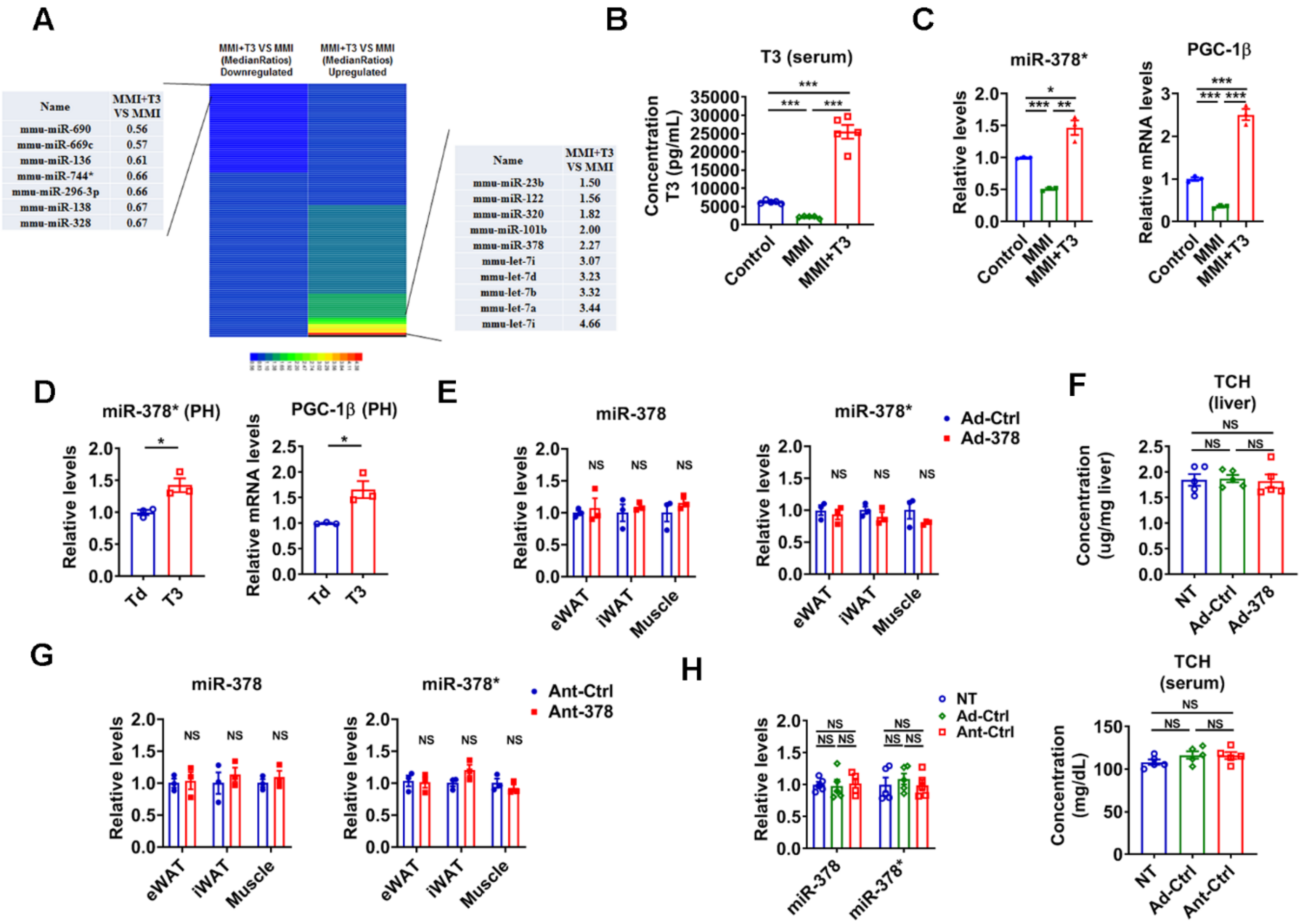

Figure S1. TH-responsive hepatic miR-378 modulates serum cholesterol levels, related to Figure 1. (A) Heatmap of differentially expressed microRNAs in the liver between hypothyroid mice (MMI) and hypothyroid mice after administration of a single dose of T3 (MMI+T3). Differentially expressed microRNAs (fold change ≥ 1.5) with name and fold change are shown. (B) Serum levels of T3 in mice under different thyroid status (control: euthyroid; MMI: hypothyroid; MMI+T3: hyperthyroid) (n = 5). (C) Relative levels of miR-378* and PGC1-β mRNA in the liver of euthyroid (control), hypothyroid (MMI), and hyperthyroid (MMI+T3) mice (n = 3). (D) Relative levels of miR-378* and PGC1-β mRNA in primary murine hepatocytes (PHs) in the absence or presence of T3 (n = 3) (Td, T3 deficient). (E) Relative levels of miR-378 and miR-378* in epididymal white adipose tissue (eWAT), inguinal white adipose tissue (iWAT), and gastrocnemius muscle (Muscle) of mice infected with Ad-Ctrl or Ad-378 (n = 3). (F) The total cholesterol levels in the liver of wild-type mice without any treatment (NT) or infected with Ad-Ctrl or Ad-378 (n = 5). (G) Relative levels of miR-378 and miR-378* in epididymal white adipose tissue (eWAT), inguinal white adipose tissue (iWAT), and gastrocnemius muscle (Muscle) of mice treated with Ant-Ctrl or Ant-378 (n = 3). (H) Relative levels of hepatic miR-378 and miR-378*, and serum total cholesterol levels of wild-type mice without any treatment (NT) or administrated with Ad-Ctrl or Ant-Ctrl (n = 5). Means ± SEM are shown. *p < 0.05; **p < 0.01; ***p < 0.001; NS, not significant.

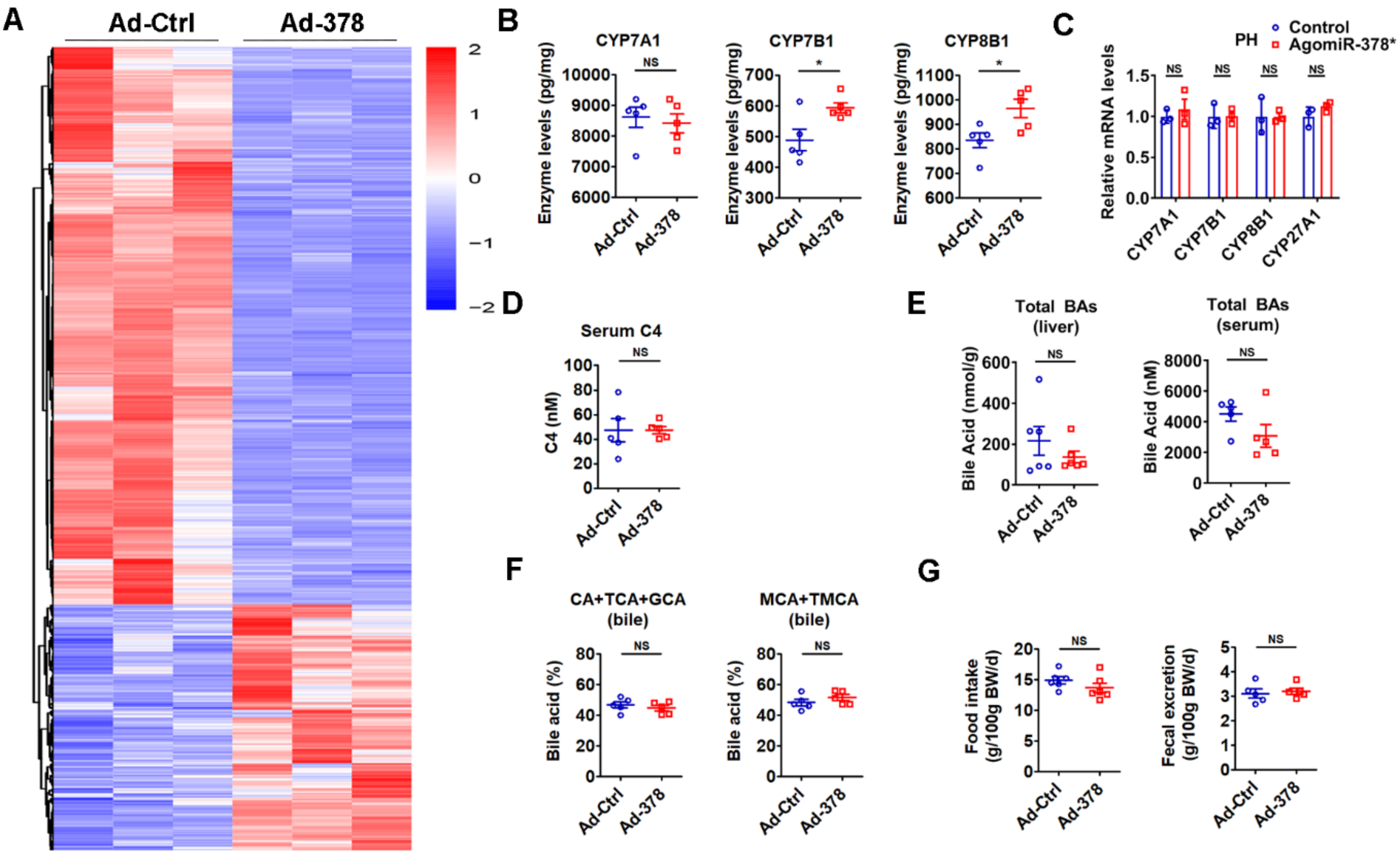

Figure S2. The effect of hepatic miR-378 on cholesterol and BA metabolism, related to Figure 2.
(A) Heatmap of all differentially expressed genes (Fold change > 2 and FDR < 0.1) identified by RNA-seq in the liver of mice infected with Ad-378 (n = 3). (B) ELISA analysis of enzyme levels of key enzymes of BA synthetic pathway in the liver of mice infected with Ad-378 (n = 5). (C) Relative mRNA levels of key enzymes of BA synthetic pathway in the PHs transfected with AgomiR-378* (n = 3). (D) The serum C4 levels of mice infected with Ad-378 (n = 5). (E) The levels of total BAs in the liver and serum of mice infected with Ad-378 (n = 5). (F) BA composition in the bile of mice infected with Ad-378 as indicated (n = 5). (G) Food intake (left) and feces output of mice (right) infected with Ad-378 (n = 5-6). Means ± SEM are shown. *p < 0.05; **p < 0.01; ***p < 0.001; NS, not significant.

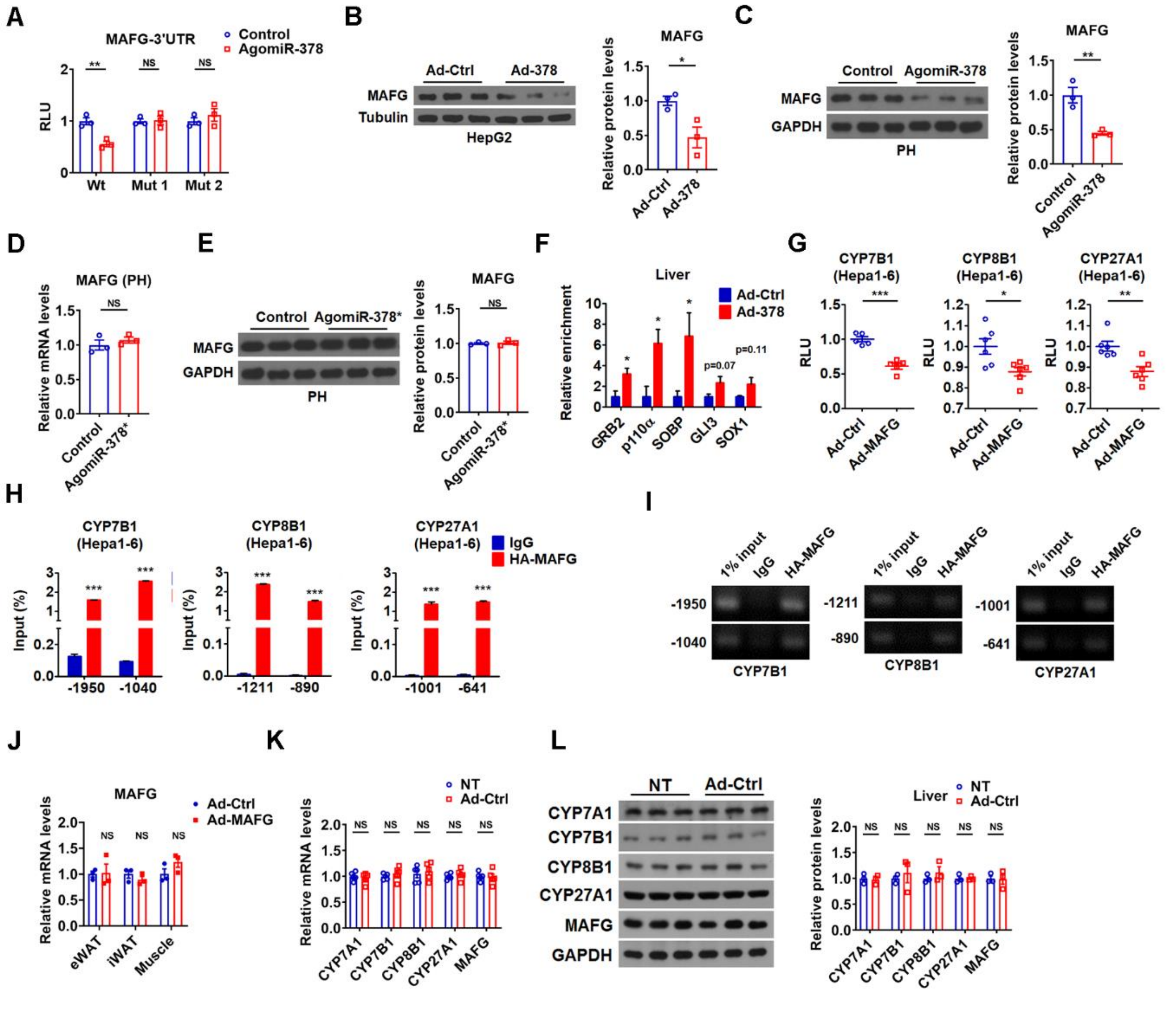

Figure S3. MAFG is a miR-378 direct target and regulates the transcription of key enzymes in BA synthetic pathway, related to Figure 3.

(A) Relative luciferase activity of the reporters containing the 3'UTR of MAFG with (Mut1, Mut2) and without (Wt) mutation as indicated in HEK293T cells transfected with AgomiR-378 ($n = 3$). (B) Western blot and densitometry analysis of MAFG in HepG2 cells infected with Ad-378. (C) Western blot and densitometry analysis of MAFG in primary murine hepatocytes (PHs) transfected with AgomiR-378. (D and E) Relative mRNA levels (D, $n = 3$), western blot and densitometry analysis (E) of MAFG in the PHs transfected with AgomiR-378*. (F) Relative levels of known or predicted miR-378 targets in Ago-bound miRNA/mRNA complexes from the liver of mice infected with Ad-378 ($n = 4$). (G) Relative luciferase activity of the reporters containing proximal CYP7B1, CYP8B1, and CYP27A1 promoters in Hepa1-6 cells infected with Ad-MAFG ($n = 5$-6). (H and I) ChIP analysis of the recruitment of MAFG to the proximal promoter regions of CYP7B1, CYP8B1, and CYP27A1 as indicated in Hepa1-6 cells transfected with HA-MAFG (H). qPCR products were analyzed by agarose gel electrophoresis as indicated (I). (J) Relative mRNA levels of MAFG in epididymal white adipose tissue (eWAT), inguinal white adipose tissue (iWAT), and gastrocnemius muscle (Muscle) of mice infected with Ad-Ctrl or Ad-MAFG ($n = 3$). (K) Relative mRNA levels of MAFG and key enzymes of BA synthetic pathway in the liver of mice without any treatment (NT) or infected with Ad-Ctrl ($n = 5$). (L) Western blot and densitometry analysis of MAFG and key enzymes of BA synthetic pathway in the liver of mice without any treatment (NT) or infected with Ad-Ctrl. Means ± SEM are shown. *$p < 0.05$; **$p < 0.01$; ***$p < 0.001$; NS, not significant.

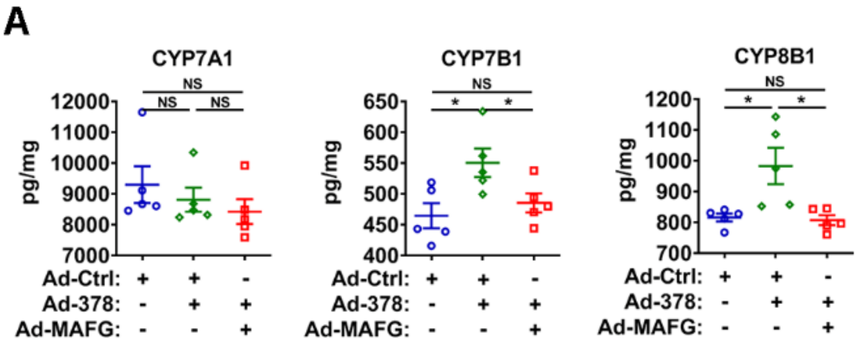

Figure S4. The effect of hepatic MAFG overexpression on miR-378-regulated cholesterol and BA metabolism, related to Figure 4.

(A) ELISA analysis of enzyme levels of key enzymes of BA synthetic pathway in the liver of mice infected with Ad-378 and Ad-MAFG as indicated (n = 5). Means ± SEM are shown. $*p < 0.05$; NS, not significant.

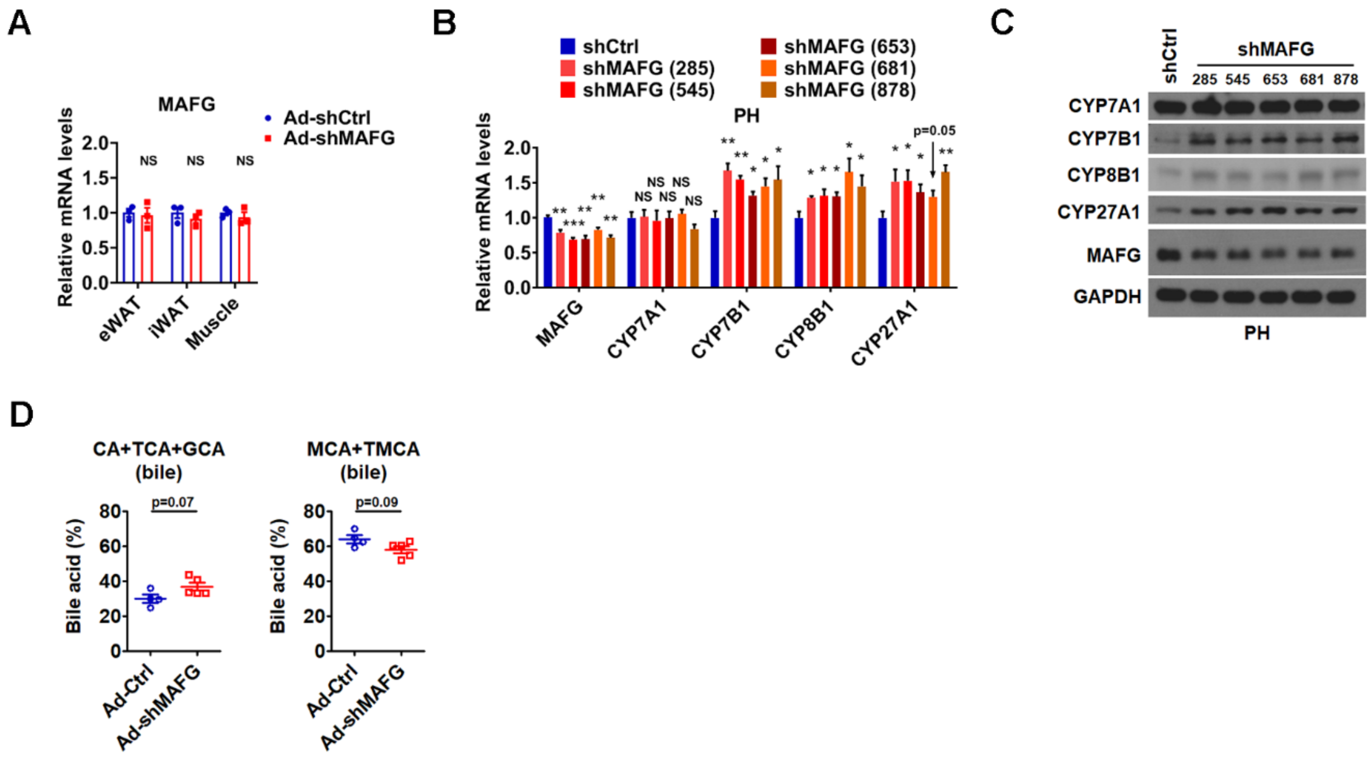

Figure S5. The effect of hepatic MAFG on cholesterol and BA metabolism, related to Figure 5.
(A) Relative mRNA levels of MAFG in epididymal white adipose tissue (eWAT), inguinal white adipose tissue (iWAT), and gastrocnemius muscle (Muscle) of mice infected with Ad-shCtrl or Ad-shMAFG (n = 3). (B and C) Relative mRNA levels (B, n=4) and western blot analysis (C) of MAFG and key enzymes of BA synthetic pathway in the PHs transfected with shRNA plasmids targeting different regions of MAFG. (D) BA composition in the bile of mice infected with Ad-shMAFG as indicated (n = 4-5). Means ± SEM are shown. *p < 0.05; **p < 0.01; ***p < 0.001; NS, not significant.

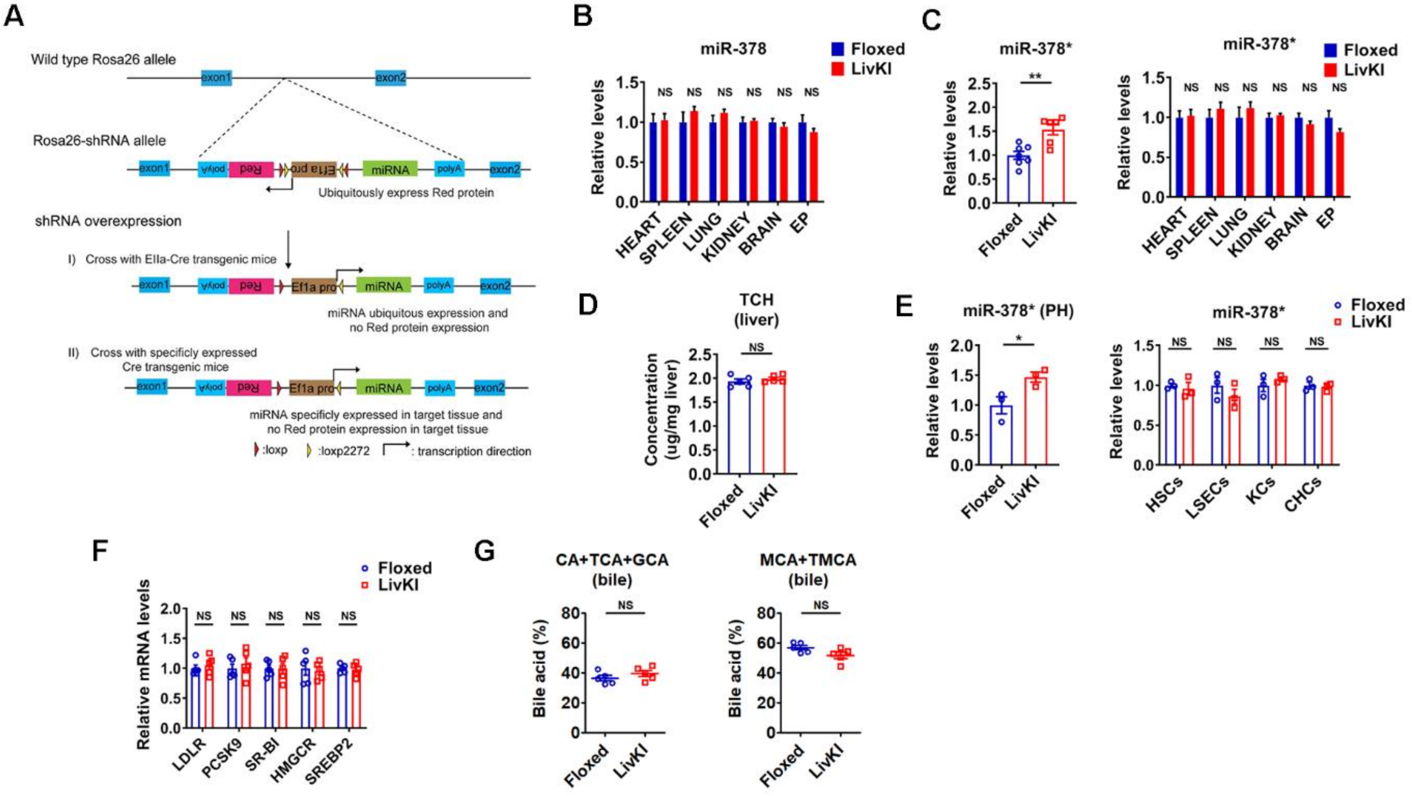

Figure S6. The effect of moderate overexpression of hepatic miR-378 on cholesterol and BA metabolism, related to Figure 6.

(A) Schematic diagram of generating a conditional miR-378/miR-378* transgenic mouse line that permits conditional expression of miR-378/miR-378* in a Cre-recombinase-dependent manner. (B) Relative levels of miR-378 in the various tissues of Floxed and LivKI mice (n = 5). (C) Relative levels of miR-378* in the liver (n = 6-7) or other tissues (n = 5) of Floxed and LivKI mice. (D) Total cholesterol (TCH) levels in the liver of Floxed and LivKI mice (n = 5). (E) Relative levels of miR-378* in the primary murine hepatocytes (PHs), cholangiocytes (CHCs), hepatic stellate cells (HSCs), liver sinusoidal endothelial cells (LSECs), and Kupffer cells (KCs) from Floxed and LivKI mice (n = 3). (F) Relative mRNA levels of genes related to cholesterol uptake (LDLR, PCSK9 and SR-BI) and cholesterol *de novo* synthesis (HMGCR and SREBP2) in the liver of Floxed and LivKI mice (n = 5). (G) BA composition in the bile of Floxed and LivKI mice (n = 5). Means ± SEM are shown. *p < 0.05; **p < 0.01; ***p < 0.001; NS, not significant.

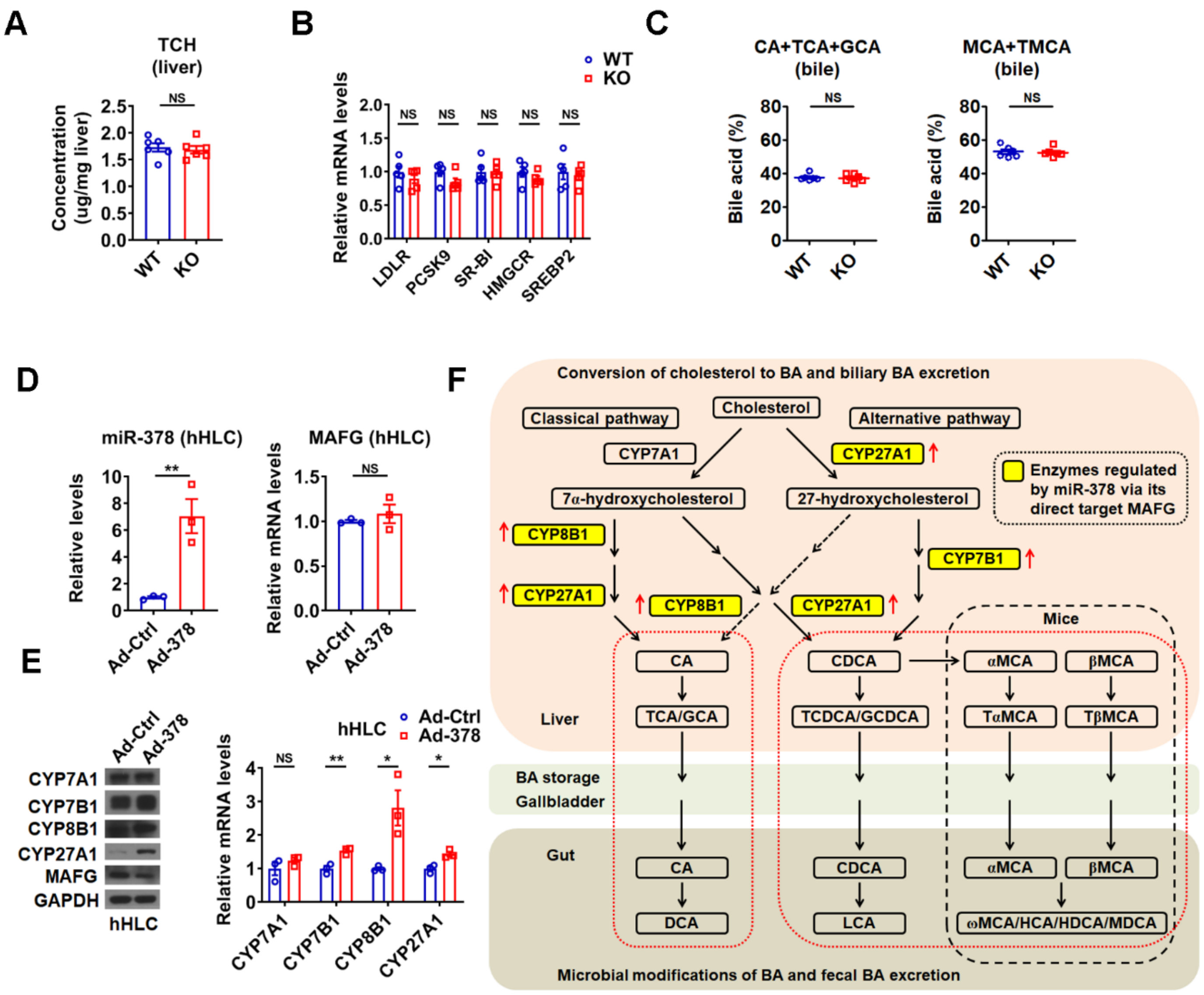

Figure S7. The effect of loss of miR-378 on cholesterol and BA metabolism, related to Figure 7.
(A) Total cholesterol levels in the liver of WT and KO mice (n = 5). (B) Relative mRNA levels of genes related to cholesterol uptake (LDLR, PCSK9 and SR-BI) and cholesterol *de novo* synthesis (HMGCR and SREBP2) in the liver of WT and KO mice (n = 5). (C) BA composition in the bile of WT and KO mice as indicated (n = 6). (D) Relative levels of miR-378 and MAFG mRNA in human hepatocyte-like cells (hHLCs) infected with Ad-378 (n = 3). (E) Representative western blot and relative mRNA levels of MAFG and key enzymes of BA synthetic pathway in hHLCs infected with Ad-378. (F) Schematic diagram of the working model of the role of miR-378 in the cholesterol and BA homeostasis. Means ± SEM are shown. *p < 0.05; **p < 0.01; ***p < 0.001; NS, not significant.

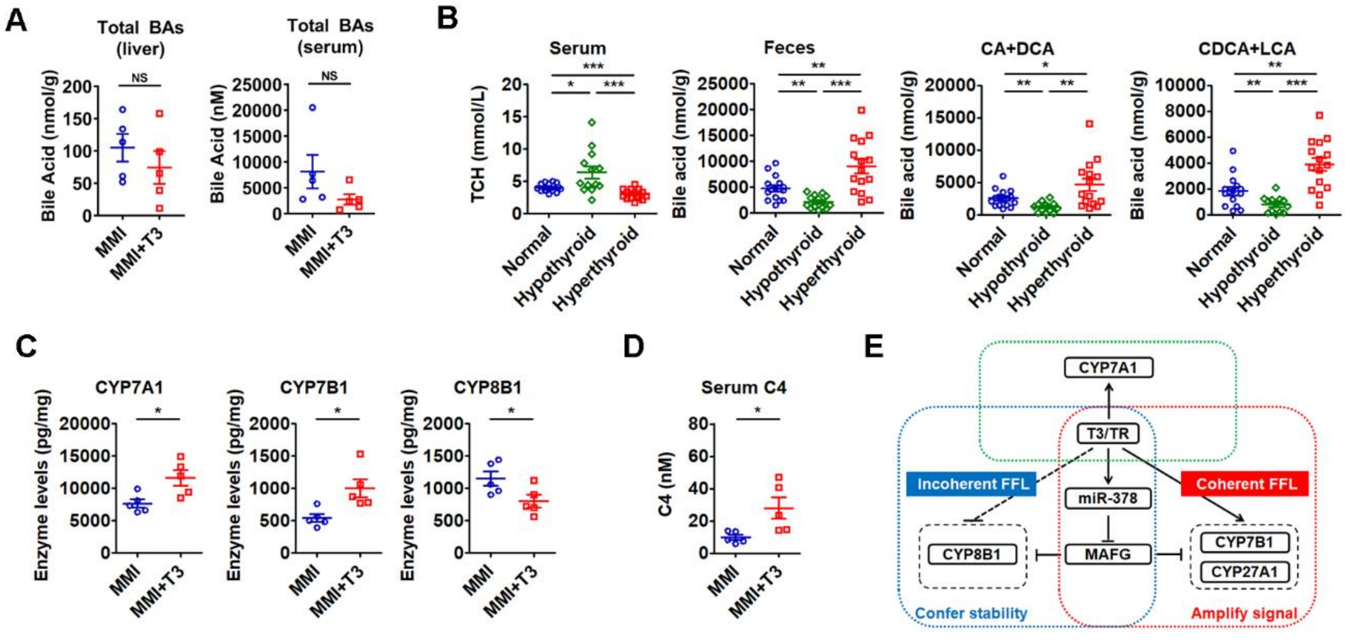

Figure S8. miR-378 is involved in the regulation of BA and cholesterol metabolism by TH, related to Figure 8.

(A) The levels of total BAs in the liver and serum of hypothyroid (MMI) and hyperthyroid (MMI+T3) mice as indicated (n = 5). (B) The levels of serum TCH, total BAs and major BAs in the feces of normal, hypothyroid, and hyperthyroid human subjects (n = 13-15). (C) ELISA analysis of enzyme levels of key enzymes of BA synthetic pathway in the liver of hypothyroid (MMI) and hyperthyroid (MMI+T3) mice (n = 5). (D) The serum C4 levels of hypothyroid (MMI) and hyperthyroid (MMI+T3) mice (n = 5). (E) Schematic diagram of the possible role of miR-378 in TH-regulated BA synthetic pathway. MiR-378 participates in either incoherent feed-forward loop (FFL) or coherent FFL to confer robust and precise controls on BA synthesis in response to TH signalling. Means ± SEM are shown. *$p < 0.05$; **$p < 0.01$; ***$p < 0.001$; NS, not significant.

Table S1. Microarray analysis of microRNA expression profile in the liver of hypothyroid mice before (MMI) and after T3 treatment (MMI+T3).

Name	MMI+T3 VS MMI MedianRatios	Name	MMI+T3 VS MMI MedianRatios
mmu-miR-690	0.56	mmu-miR-466a-3p	1.00
mmu-miR-669c	0.57	mmu-miR-34c*	1.00
mmu-miR-136	0.61	mmu-miR-743a	1.00
mmu-miR-744*	0.66	mmu-miR-291a-5p	1.00
mmu-miR-296-3p	0.66	mmu-miR-466b-5p	1.00
mmu-miR-138	0.67	mmu-miR-881	1.01
mmu-miR-328	0.67	mmu-miR-465b-5p	1.01
mmu-miR-467c	0.69	mmu-miR-423-3p	1.01
mmu-miR-106b	0.70	mmu-miR-184	1.01
mmu-miR-705	0.71	mmu-miR-22*	1.01
mmu-miR-144	0.73	mmu-miR-503	1.02
mmu-miR-192	0.74	mmu-miR-17*	1.02
mmu-miR-721	0.75	mmu-miR-34b-3p	1.02
mmu-miR-709	0.75	mmu-miR-673-3p	1.02
mmu-miR-29b*	0.75	mmu-miR-21*	1.02
mmu-miR-208	0.76	mmu-miR-710	1.02
mmu-miR-666-5p	0.76	mmu-miR-197	1.03
mmu-miR-686	0.76	mmu-miR-877	1.03
mmu-miR-195	0.77	mmu-miR-466g	1.03
mmu-miR-669b	0.77	mmu-miR-574-3p	1.03
mmu-miR-467b	0.77	mmu-miR-466c-5p	1.03
mmu-miR-124	0.77	mmu-miR-382*	1.03
mmu-miR-742	0.78	mmu-miR-490	1.04
mmu-let-7e	0.78	mmu-miR-433*	1.04
mmu-miR-298	0.78	mmu-miR-695	1.04
mmu-miR-342-5p	0.79	mmu-miR-290-3p	1.04
mmu-miR-341	0.79	mmu-miR-138*	1.04
mmu-miR-883b-3p	0.79	mmu-miR-471	1.04
mmu-miR-297b-5p	0.79	mmu-miR-467b*	1.04
mmu-miR-382	0.80	mmu-miR-883b-5p	1.04
mmu-miR-183	0.80	mmu-miR-326	1.05
mmu-miR-497	0.80	mmu-miR-667	1.05
mmu-miR-99b	0.81	mmu-miR-466b-3-3p	1.05
mmu-miR-674	0.81	mmu-miR-582-3p	1.05
mmu-miR-212	0.82	mmu-miR-875-3p	1.05
mmu-miR-297c	0.82	mmu-miR-872*	1.06
mmu-miR-491	0.82	mmu-miR-351	1.06
mmu-miR-335-5p	0.82	mmu-miR-381	1.06
mmu-miR-546	0.82	mmu-miR-423-5p	1.06
mmu-miR-871	0.83	mmu-miR-24-1*	1.06
mmu-miR-20a	0.83	mmu-miR-503*	1.07
mmu-miR-323-3p	0.84	mmu-miR-697	1.07
mmu-miR-34c	0.84	mmu-miR-15b	1.08

mmu-miR-376c	0.84	mmu-miR-103	1.08
mmu-miR-675-5p	0.84	mmu-miR-9*	1.08
mmu-miR-671-5p	0.86	mmu-miR-322	1.08
mmu-miR-297a	0.86	mmu-miR-465c-5p	1.08
mmu-miR-670	0.86	mmu-let-7g	1.09
mmu-miR-20b*	0.86	mmu-let-7d*	1.09
mmu-miR-330*	0.87	mmu-miR-770-3p	1.09
mmu-miR-30a	0.87	mmu-miR-542-3p	1.09
mmu-miR-679	0.88	mmu-miR-329	1.09
mmu-miR-31	0.88	mmu-miR-325	1.09
mmu-miR-16*	0.88	mmu-miR-300*	1.09
mmu-miR-452	0.88	mmu-miR-743b-5p	1.09
mmu-miR-720	0.89	mmu-miR-681	1.09
mmu-miR-218-2*	0.89	mmu-miR-185	1.10
mmu-miR-483	0.89	mmu-miR-713	1.10
mmu-miR-1	0.89	mmu-miR-494	1.10
mmu-miR-801	0.90	mmu-miR-207	1.10
mmu-miR-574-5p	0.90	mmu-miR-30c	1.11
mmu-miR-669a	0.90	mmu-miR-206	1.11
mmu-miR-467e	0.91	mmu-miR-361	1.11
mmu-miR-486	0.91	mmu-miR-150	1.11
mmu-miR-688	0.91	mmu-miR-485*	1.11
mmu-miR-141*	0.91	mmu-miR-350	1.12
mmu-miR-881*	0.92	mmu-miR-208b	1.12
mmu-miR-194	0.92	mmu-let-7c	1.13
mmu-miR-665	0.92	mmu-miR-761	1.13
mmu-miR-340-5p	0.92	mmu-miR-215	1.14
mmu-let-7c-1*	0.92	mmu-miR-24	1.14
mmu-miR-465a-5p	0.92	mmu-miR-369-3p	1.14
mmu-miR-874	0.92	mmu-miR-99b*	1.14
mmu-miR-467e*	0.92	mmu-miR-107	1.15
mmu-miR-327	0.92	mmu-miR-129-5p	1.17
mmu-miR-200b*	0.93	mmu-miR-412	1.18
mmu-miR-678	0.93	mmu-miR-26b	1.18
mmu-miR-325*	0.93	mmu-miR-711	1.19
mmu-miR-362-3p	0.93	mmu-miR-344	1.20
mmu-miR-466f-3p	0.93	mmu-miR-200b	1.22
mmu-miR-185	0.94	mmu-miR-294*	1.22
mmu-miR-466f-5p	0.94	mmu-miR-183*	1.23
mmu-miR-470	0.94	mmu-miR-130a	1.23
mmu-miR-300	0.94	mmu-miR-15a	1.26
mmu-miR-468	0.94	mmu-miR-214	1.26
mmu-miR-290-5p	0.95	mmu-miR-125a-5p	1.26
mmu-miR-466a-5p	0.95	mmu-miR-488*	1.28
mmu-miR-196a*	0.95	mmu-miR-125b-3p	1.28
mmu-miR-693-5p	0.95	mmu-miR-30c-1*	1.28
mmu-miR-200c	0.95	mmu-miR-455	1.28

mmu-miR-337-3p	0.95	mmu-miR-101a	1.28
mmu-miR-463	0.95	mmu-let-7f	1.29
mmu-miR-297b-3p	0.96	mmu-miR-16	1.29
mmu-miR-466e-5p	0.96	mmu-miR-22	1.34
mmu-miR-685	0.96	mmu-miR-29a	1.35
mmu-miR-291b-5p	0.97	mmu-miR-21	1.36
mmu-miR-34a	0.97	mmu-miR-744	1.38
mmu-miR-467a*	0.97	mmu-miR-409-5p	1.38
mmu-miR-466d-3p	0.97	mmu-let-7d	1.39
mmu-miR-142-5p	0.97	mmu-miR-345-5p	1.39
mmu-miR-200c*	0.97	mmu-miR-26a	1.39
mmu-miR-551b	0.98	mmu-miR-126-3p	1.40
mmu-miR-466d-5p	0.98	mmu-miR-130b	1.41
mmu-miR-143	0.98	mmu-miR-193	1.42
mmu-miR-30b*	0.98	mmu-miR-805	1.44
mmu-miR-714	0.98	mmu-miR-451	1.49
mmu-miR-706	0.98	mmu-miR-23b	1.50
mmu-miR-883a-5p	0.98	mmu-miR-122	1.56
mmu-miR-717	0.98	mmu-miR-320	1.82
mmu-miR-615-3p	0.99	mmu-miR-101b	2.00
mmu-miR-882	0.99	mmu-miR-378	2.27
mmu-miR-701	0.99	mmu-let-7i	3.07
mmu-miR-691	0.99	mmu-let-7d	3.23
mmu-miR-763	0.99	mmu-let-7b	3.32
mmu-miR-668	1.00	mmu-let-7a	3.44
mmu-miR-130b*	1.00	mmu-let-7i	4.66
mmu-miR-30c-2*	1.00		

Pooled samples from three mice were used.

Table S2. Sequence information for primers and oligonucleotides

Name	Sequence（5’—3’）	Notes
mCYP8B1-F	GGGAGTGGGTGGAAGTGAG	RT-PCR
mCYP8B1-R	GTCCTGCATGGATGAAGCT	RT-PCR
mCYP7B1-F	GGAGCCACGACCCTAGATG	RT-PCR
mCYP7B1-R	TGCCAAGATAAGGAAGCCAAC	RT-PCR
mCYP7A1-F	GAGAGTGAATCAGGGGACCA	RT-PCR
mCYP7A1-R	TCAGGAATGGAGGGTTTCAG	RT-PCR
mCYP27A1-F	AGGGCCTCACATCAACAGAG	RT-PCR
mCYP27A1-R	GCTGACGCTGTAGGACACAT	RT-PCR
mMAFG-F	GAGACCCAGAGGCTACAGATT	RT-PCR
mMAFG-R	AATGTACTCCACATGGGGCAA	RT-PCR
mPCSK9-F	GCTGGTTTGACTGGATGGTT	RT-PCR
mPCSK9-R	ACCTTTGGCGAGGTCTAGGT	RT-PCR
mSREBP-2-F	AGCTTGCCCGAATTGTATGTG	RT-PCR
mSREBP-2-R	TCTGTTGTGAACCATGTGACTTC	RT-PCR
mHMGCR-F	AAACAGGGAAGATCGAGCCAG	RT-PCR
mHMGCR-R	GGTCTGACCAAGCTATCAGGTT	RT-PCR
mSR-BI-F	AGTGGCCCCGAATCATTGAC	RT-PCR
mSR-BI-R	CTAACTAAACACCAGACAGAGGC	RT-PCR
mLDLR-F	AGTGGCCCCGAATCATTGAC	RT-PCR
mLDLR-R	CTAACTAAACACCAGACAGAGGC	RT-PCR
hCYP7B1-F	AAAGGTTGGCTTCCTTATCTTGG	RT-PCR
hCYP7B1-R	GCAACTGACTGATGCTAAATGCT	RT-PCR
hCYP8B1-F	ATTTGGATACCGTTCAGTGCAA	RT-PCR
hCYP8B1-R	CAGAAGCGAAAGAGGCTGTC	RT-PCR
hcCYP7A1-F	GAGAAGGCAAACGGGTGAAC	RT-PCR
hcCYP7A1-R	GGATTGGCACCAAATTGCAGA	RT-PCR
hCYP27A1-F	CGGCAACGGAGCTTAGAGG	RT-PCR
hCYP27A1-R	GGCATAGCCTTGAACGAACAG	RT-PCR
hMAFG-F	ATGACGACCCCCAATAAAGGAA	RT-PCR
hMAFG-R	TCAGGCTGGTGCCATTCTCA	RT-PCR
TBP-F	ACGGACAACTGCGTTGATTT	RT-PCR
TBP-R	TTCTTGCTGCTAGTCTGGATTG	RT-PCR
18s-F	ACCGCAGCTAGGAATAATGGA	RT-PCR
18s-R	CAAATGCTTTCGCTCTGGTC	RT-PCR
miR-378-RT	CTCAACTGGTGTCGTGGAGTCGGCAATTCAG TTGAGCTTCTGAC	stem-loop RT
miR-378*-RT	CTCAACTGGTGTCGTGGAGTCGGCAATTCAG TTGAGCACAGGAC	stem-loop RT
U6-RT	CTCAACTGGTGTCGTGGAGTCGGCAATTCAG TTGAGATATGGAA	stem-loop RT
miR-378-F	ACACTCCAGCTGGGACTGGACTTGGA	stem-loop PCR
miR-378*-F	ACACTCCAGCTGGGCTCCTGACTCCAG	stem-loop PCR
U6-F	ACACTCCAGCTGGGATTCGTGAAGCG	stem-loop PCR
Universal-R	CTCAACTGGTGTCGTGGAGTCGG	stem-loop PCR
653-F	CACCGCCACCAGCGTCATCACAATACGAATAT TGTGATGACGCTGGTGGC	shMAFG
653-R	AAAAGCCACCAGCGTCATCACAATATTCGTAT TGTGATGACGCTGGTGGC	shMAFG

681-F	CACCCCAAGACGGATGCTCGGTCATCTCGAG ATGACCGAGCATCCGTCTTGG	shMAFG
681-R	AAAACCAAGACGGATGCTCGGTCATCTCGAG ATGACCGAGCATCCGTCTTGG	shMAFG
878-F	CACCTCGTTGTCACGCCTGTGTTTCCTCGAG GAAACACAGGCGTGACAACGA	shMAFG
878-R	AAAATCGTTGTCACGCCTGTGTTTCCTCGAGG AAACACAGGCGTGACAACGA	shMAFG
285-F	CACCTGACCGACGAGGAGCTGGTAACTCGAG TTACCAGCTCCTCGTCGGTCA	shMAFG
285-R	AAAATGACCGACGAGGAGCTGGTAACTCGAG TTACCAGCTCCTCGTCGGTCA	shMAFG
545-F	CACCTACGAGGCCCTGCAGAACTTTCTCGAG AAAGTTCTGCAGGGCCTCGTA	shMAFG
545-R	AAAATACGAGGCCCTGCAGAACTTTCTCGAGA AAGTTCTGCAGGGCCTCGTA	shMAFG
mMAFG-F	CAGGCGGCCAGTCCTCCTAA	adenovirus
mMAFG-R	CAACTCTGCCACGCACCTAG	adenovirus
mMAFG-UTR-F	CTAGGTGCGTGGCAGAGTTG	luciferase assay
mMAFG-UTR-R	AGACCACCAGGGAGACAAGG	luciferase assay
Mut1-F	CGAGAGTGGGCGCTGTGGGAGTGGA	luciferase assay
Mut2-F	GGAGAGTGGGCGCTGTGGGAGTGGA	luciferase assay
Mut Uni-R	ACTCCTAGCCTGCACGGCCTTGGTA	luciferase assay
mPGC1B(-329)-F	GCCCAGCGCTAACGCAACGTGA	ChIP-PCR
mPGC1B(-329)-R	AGGTGGCGGTGCCCGGGCCA	ChIP-PCR
mPGC1B(-951)-F	AGCAAGGACAGAGTGACTCAG	ChIP-PCR
mPGC1B(-951)-R	CAACCTTGTAAGTACCAACCCA	ChIP-PCR
mCYP8B1-1211bp-F	GCCAGACACTGTCCTAAGAGC	ChIP-PCR
mCYP8B1-1211bp-R	GAGTACACCCATGTCAGTGATTTT	ChIP-PCR
mCYP8B1-890bp-F	CAGGGAGAGATCCTGACTCAA	ChIP-PCR
mCYP8B1-890bp-R	GGGTTCTCTGGAAAAGCAGA	ChIP-PCR
mCYP27A1-1001-F	GTCTGAAGCCAGGCCATGTA	ChIP-PCR
mCYP27A1-1001-R	TGCAAGTGGCACACAGACAA	ChIP-PCR
mCYP27A1-641-F	TTAGAGGCTTGCGCAACCAT	ChIP-PCR
mCYP27A1-641-R	TGAAGACGTGTAGGCCAGGC	ChIP-PCR
mCYP7A1-951-F	CTGTCTTGTTAGTACCTGAA	ChIP-PCR
mCYP7A1-951-R	GGCAAGCACTCTACCGCTGA	ChIP-PCR
mCYP7A1-561-F	ACATGAAGCACCTGTGGCTT	ChIP-PCR
mCYP7A1-561-R	GGATGTGAACGGTGTGGAAC	ChIP-PCR
mCYP7B1-1950-F	GCAGTAGAGGTTGCTGGTCA	ChIP-PCR
mCYP7B1-1950-R	TATGGACCAGAACTGGCCCT	ChIP-PCR
mCYP7B1-1040-F	TGTCCAATGCTGACCTCTTG	ChIP-PCR
mCYP7B1-1040-R	AAGAGACCACTGACCTGCAT	ChIP-PCR
mCYP7A1-F	GGGGTACCTGCACAAAGCATTAGGAGGT	promoter cloning
mCYP7A1-R	CCGCTCGAGTCTAGTCAGTTCATGTCACA	promoter cloning
mCYP7B1-F	GGGGTACCCTAGTCACGTCTGGTTCTAG	promoter cloning
mCYP7B1-R	CCGCTCGAGAGAAGTCTTTATTAGGACAT	promoter cloning
mCYP8B1-F	GGGGTACCTGTTGTACGACAGAAAGTAT	promoter cloning
mCYP8B1-R	CCGCTCGAGAACACCTTGAGCACCAGTTC	promoter cloning
mCYP27A1-F	GGGGTACCAGTCGAGTTATTTGAAATCA	promoter cloning
mCYP27A1-R	CCGCTCGAGAGTGCCCAGACCCAGGTGGC	promoter cloning

GLI3-F	AGTGTTCTATCCCAAGATTT	RIP-PCR
GLI3-R	TAAAGAGATCTCATGTCCCA	RIP-PCR
P110a-F	AAGGCTGAACTCAGTCTTGA	RIP-PCR
P110a-R	CGACATGATCTGTTTACAGT	RIP-PCR
MAFG-F	CAACAGTGATGTTGGGTTGC	RIP-PCR
MAFG-R	AAGACCAGTACACTTGCCCA	RIP-PCR
Grb2-F	TGTCTTATTTCAAGGGTGTT	RIP-PCR
Grb2-R	TATTTACACAGGATAGGAGA	RIP-PCR
SOX1-F	AATCATGACATTAGAATGAA	RIP-PCR
SOX1-R	AATGGCTAGTCCATATTCAT	RIP-PCR
SOBP-F	TTGTGTGCACTTGTACAGTT	RIP-PCR
SOBP-R	CTGTACGACTGTCTCAGCGA	RIP-PCR

Table S3. The density of specific bands from immunoblots and normalization.

Figure 2F

	Ad-Ctrl		Ad-378	
CYP7A1	1.00	1.02	1.10	0.99
CYP7B1	1.00	0.80	1.72	1.56
CYP8B1	1.00	0.83	1.58	1.44
CYP27A1	1.00	1.15	1.53	1.50
GAPDH	1.00	1.13	1.14	1.14

Figure 2G

	Ant-Ctrl		Ant-378	
CYP7A1	1.00	0.96	1.03	0.93
CYP7B1	1.00	1.20	0.66	0.56
CYP8B1	1.00	0.84	0.56	0.54
CYP27A1	1.00	1.17	0.65	0.80
GAPDH	1.00	0.99	0.94	1.09

Figure 3C

	NT	Ad-Ctrl	Ad-378
MAFG	1.00	0.90	0.53
GAPDH	1.00	0.97	1.09

Figure 3D

	NT	Ant-Ctrl	Ant-378
MAFG	1.00	1.10	1.71
GAPDH	1.00	1.17	0.88

Figure 3H

	Ad-Ctrl		Ad-MAFG	
CYP7A1	1.00	1.10	1.14	1.05
CYP7B1	1.00	0.70	0.45	0.16
CYP8B1	1.00	0.90	0.48	0.53
CYP27A1	1.00	1.07	0.81	0.64
MAFG	1.00	0.48	2.15	2.60
GAPDH	1.00	1.06	1.05	1.01

Figure 4A

	Ad-Ctrl	Ad-Ctrl+Ad-378	Ad-378+Ad-MAFG
MAFG	1.00	0.28	1.08
actin	1.00	0.99	1.03

Figure 4F

	Ad-Ctrl		Ad-Ctrl+Ad-378		Ad-378+Ad-MAFG	
CYP7A1	1.00	1.03	1.06	1.01	1.08	1.10
CYP7B1	1.00	0.91	1.30	1.26	0.58	0.24
CYP8B1	1.00	1.11	2.06	2.07	1.11	0.90
CYP27A1	1.00	1.07	1.39	1.35	0.90	0.89

MAFG	1.00	0.87	0.48	0.61	1.49	1.37
GAPDH	1.00	0.91	1.05	1.06	1.07	0.95

Figure 5F

	Ad-shCtrl		Ad-shMAFG	
MAFG	1.00	1.16	0.80	0.63
GAPDH	1.00	0.99	1.14	1.31

Figure 5H

	Ad-shCtrl		Ad-shMAFG	
CYP7A1	1.00	1.03	0.92	0.81
CYP7B1	1.00	1.04	2.37	2.66
CYP8B1	1.00	0.97	1.57	1.35
CYP27A1	1.00	1.00	1.67	1.53
GAPDH	1.00	1.11	0.97	0.63

Figure 8G

	WT-MMI			WT-MMI+T3			KO-MMI+T3		
CYP7A1	1.00	1.33	1.01	2.54	2.35	2.46	2.58	2.58	2.43
CYP7B1	1.00	0.91	0.69	3.32	3.78	3.87	2.04	2.21	1.84
CYP8B1	1.00	0.95	1.08	0.52	0.54	0.47	0.34	0.39	0.35
CYP27A1	1.00	1.01	1.05	4.01	4.23	4.57	2.71	2.63	2.26
GAPDH	1.00	1.21	1.11	1.02	0.94	0.83	0.85	0.85	0.89

Figure S5C

		shMAFG				
	shCtrl	sh285	sh545	sh653	sh681	sh878
CYP7A1	1.00	1.01	1.01	0.98	0.95	0.94
CYP7B1	1.00	2.95	2.22	2.38	2.07	2.80
CYP8B1	1.00	1.55	1.70	1.54	1.23	1.35
CYP27A1	1.00	1.51	1.76	1.75	1.55	1.42
MAFG	1.00	0.64	0.65	0.62	0.61	0.65
GAPDH	1.00	0.91	1.02	0.90	1.02	0.95
